# Comparative Safety of Glucagon‐Like Peptide 1 Receptor Agonists (GLP‐1‐RAs) in Type 2 Diabetes and Chronic Weight Management: A Real‐World Data Study

**DOI:** 10.1002/pds.70214

**Published:** 2025-09-14

**Authors:** Sarah Ruth Hurwitz, Stephan Lanes, Tracey Quimbo, Anahit Papazian, Jeff White, Vicki Fisher, Mark J. Cziraky, Matthew J. Crowley, Vincent J. Willey

**Affiliations:** ^1^ Carelon Research Wilmington Delaware USA; ^2^ CarelonRx Morristown New Jersey USA; ^3^ Duke University Medical Center Durham North Carolina USA

**Keywords:** diabetes, GLP‐1‐RA, naltrexone‐bupropion, phentermine‐topiramate, safety, SGLT2, weight management

## Abstract

**Purpose:**

This study assessed serious clinical outcomes comparing glucagon‐like peptide 1 receptor agonists (GLP‐1‐RAs) with sodium glucose co‐transporter 2 inhibitors (SGLT2‐Is) in patients with type 2 diabetes (T2DM) and patients without diabetes using two chronic weight management (CWM) regimens.

**Methods:**

We performed a new user, active comparator cohort study in a large, national U.S. claims database. Adults who initiated GLP‐1‐RAs, SGLT2‐Is, naltrexone/bupropion (NalBup), or phentermine/topiramate (PhenTop) from 1 January 2016 to 31 December 2023 were included. Potential confounding was controlled using propensity score weighting for 82 clinical and demographic covariates, and risk ratios (RRs) were estimated.

**Results:**

This study included 330,684 GLP‐1‐RA users and 264,277 SGLT2‐I users with T2DM. Among CWM patients without diabetes, we studied over 25,000 GLP‐1‐RA users, 5019 NalBup users, and 3841 PhenTop users. In both indications, GLP‐1‐RA users had higher rates of hospitalizations for gallbladder and biliary diseases with RRs ranging from 1.14 (95% CI: 1.06–1.22) in T2DM patients to 3.32 (95% CI: 1.44–7.64) in CWM patients. No reduction in the rate of cardiovascular events was observed for GLP‐1‐RA users with RRs ranging from 0.92 (95% CI: 0.37–2.25) in CWM patients to 1.03 (95% CI: 0.99–1.08) in T2DM patients. In T2DM patients, GLP‐1‐RA users had a lower rate of acute liver injury (RR: 0.76; 95% CI: 0.64–0.91).

**Conclusions:**

This study corroborates an increased risk of hospitalization for gall bladder and biliary conditions among users of GLP‐1‐RAs and found similar rates as comparators of MI or stroke when GLP‐1‐RAs were used for T2DM or CWM. This real‐world study complements placebo‐controlled trials and can further inform prescribing decisions.

**Protocol Registration:** The study protocol was pre‐registered at the Center for Open Science's Real‐World Evidence Registry and is publicly accessible online (https://doi.org/10.17605/OSF.IO/PSY74).

**Summary**

- Dramatic increases in the use of glucagon‐like peptide‐1 receptor agonists (GLP‐1‐RAs) underscore the importance of high‐quality safety evidence to inform decision‐making.
- We used the target trial emulation framework to compare the incidence of serious clinical outcomes in new users of GLP‐1‐RAs versus new users of sodium glucose co‐transporter 2 inhibitors for type 2 diabetes (T2DM), and versus naltrexone/bupropion or phentermine/topiramate for chronic weight management (CWM).
- Hospitalizations for gall bladder and biliary conditions were more common among GLP‐1‐RA users versus comparator users.
- MI or stroke rates were similar across therapies in these real‐world cohorts.

## 1 Introduction

Glucagon‐like peptide 1 receptor agonists (GLP‐1‐RAs) have been available in the United States for the treatment of type 2 diabetes (T2DM) for almost 20 years [1]. However, GLP‐1‐RA prescribing has increased dramatically in recent years due to revised prescribing guidance in T2DM [2, 3, 4, 5] and new chronic weight management (CWM) indications [6, 7, 8].

The safety profile of GLP‐1‐RAs has been described largely in individuals with T2DM participating in randomized, placebo‐controlled clinical trials. Randomized placebo‐controlled clinical trials provide important evidence of safety and effectiveness and are the backbone of regulatory approval. However, clinical trials have not offered a comprehensive assessment of the incidence of serious clinical events comparing current GLP‐1‐RA therapies with therapeutic alternatives for both T2DM and CWM in a real‐world setting.

Recent data regarding long‐term exposure in large, real‐world populations including individuals without T2DM raises new questions about the risk–benefit assessment [9, 10]. Increased risks of gastrointestinal adverse events have been reported in patients with T2DM, as well as adverse events associated with mental health, hepatic injury, and thyroid cancer [9, 11, 12, 13, 14, 15, 16]. In addition, beneficial effects of some GLP‐1 RAs on cardiovascular outcomes have been observed in both T2DM and obese populations [17, 18, 19], as well as potential benefits in metabolic dysfunction‐associated fatty liver disease and metabolic dysfunction‐associated steatohepatitis [20, 21].

With more widespread use of GLP‐1‐RAs, there is a growing need to better inform risk–benefit decisions about these therapies. This study compared new users of GLP‐1‐RAs to new users of other prescription medications, separately in patients with T2DM and CWM indications. We estimated the incidence of severe gastrointestinal events, thyroid cancer, acute liver injury, psychiatric hospitalization, suicidal ideation and self‐harm, myocardial infarction (MI) and stroke, and all‐cause mortality during the first 6 months of treatment.

## 2 Methods

### 2.1 Study Design

We designed this retrospective cohort study to emulate a target trial of GLP‐1‐RA use compared to active comparator treatments [22]. We conducted a new user, active comparator cohort study in the Healthcare Integrated Research Database (HIRD). Analyses were conducted separately for patients being treated for T2DM (T2DM cohort) and two cohorts of patients treated for chronic weight management (CWM). The study protocol was pre‐registered at the Center for Open Science's Real‐World Evidence Registry and is publicly accessible [23].

### 2.2 Data Sources

The data presented in this report are from U.S. patients with commercial health insurance or Medicare Advantage appearing in the HIRD. The HIRD is a large healthcare database maintained by Carelon Research for use in health outcomes and pharmacoepidemiologic research. The study utilized data from the HIRD, which contains medical and pharmacy claims from commercially insured/Medicare Advantage health plan members across the U.S. and electronic health record (EHR) data for a subset of individuals. Mortality data are included in the HIRD and are ascertained from multiple sources including hospital discharge status, disenrollment records, death date from the Center for Medicare and Medicaid Services, utilization management claims, National Technical Information Service Death Master File data from the Social Security Administration, and obituaries. This study utilized HIRD data from January 01, 2006 to December 31, 2023.

### 2.3 Study Population

In the T2DM cohort, patients initiating GLP‐1‐RAs were compared to those initiating sodium glucose co‐transporter 2 inhibitors (SGLT2‐Is), and in the two CWM cohorts, patients initiating GLP‐1‐RAs were compared to those initiating either (1) combination naltrexone hydrochloride/bupropion hydrochloride (NalBup) or (2) combination phentermine/topiramate extended‐release (PhenTop).

Patients were included in the T2DM cohort if they (1) had at least one dispensing of a GLP‐1‐RA or SGLT2‐I on or after January 01, 2016, (2) had at least 6 months of continuous enrollment with medical and pharmacy benefits prior to their first claim for a study drug (index date), (3) were aged 18 years or older on their index date, (4) had a diagnosis code for T2DM on or before the index date, and (5) had no prior claims for GLP‐1‐RAs or SGLT2‐Is in the HIRD.

Patients were initially included in the CWM cohorts if they had (1) at least one dispensing of a GLP‐1‐RA, NalBup, or PhenTop on or after January 01, 2016, (2) at least 6 months of continuous enrollment with medical and pharmacy benefits prior to their first claim for a study drug (index date), (3) were aged 18 years or older on the index date, (4) had an obesity diagnosis on or within 6 months before the index date, or an overweight diagnosis on or 6 months before the index date with, as a high‐risk indicator, a diagnosis of hypertension or dyslipidemia on or before the index date, (5) had no prior exposure to GLP‐1‐RAs, and no prior exposure to NalBup (for the comparison of GLP‐1‐RAs to naltrexone/bupropion) or no prior exposure to PhenTop (for the comparison of GLP‐1‐RAs to phentermine/topiramate).

After examining baseline covariates, the CWM cohorts were further restricted to commercially insured patients with an index date on or after January 01, 2020, who had no diagnoses of T2DM, type 1 diabetes, or prediabetes, or prescription fill for any diabetes drug. These restrictions were made because only commercially insured patients could receive the comparator medications due to health plan restrictions, and there was noncomparability [24] of GLP‐1‐RA users and comparators before 2020 or when diabetes patients were included. After starting follow‐up after January 2020 and excluding patients with diabetes or prediabetes, covariates were balanced between GLP‐1‐RA users and comparators. The “Statistical Analysis” section provides additional information on propensity score modeling and assessment of covariate balance.

### 2.4 Exposures

Patients were considered exposed to a study drug if they had at least one pharmacy claim for a dispensing for GLP‐1‐RAs, sodium glucose co‐transporter 2 inhibitors (SGLT2‐Is), naltrexone hydrochloride/bupropion hydrochloride (NalBup), or phentermine/topiramate extended‐release (PhenTop). The index date for each patient was the date of their first (index) exposure recorded in the database during the intake period (January 01, 2016 through December 31, 2023 for the T2DM cohort and January 01, 2020 through December 31, 2023 for the weight loss cohorts). At least one study drug from each comparison group was on the market during the follow‐up periods. To allow for nonadherence and washout, subsequent dispensings were classified as continuous exposure if they occurred within 1.5 times the days' supply of the previous dispensing.

### 2.5 Follow‐Up

Follow‐up for all outcomes began on the day after the start of therapy and continued until the first occurrence of the outcome being analyzed, pregnancy, death, health plan disenrollment, malignancy, weight loss surgery, discontinuation of the study drug, initiation of a comparator treatment, or December 31, 2023 (last day of available data at the time of analysis).

### 2.6 Outcomes

Eleven outcomes were selected based on published literature for GLP‐1‐RAs: gastrointestinal hospitalizations (including gastroparesis, bowel obstruction, and other diagnoses [23]), gastroparesis hospitalizations, bowel obstruction hospitalizations, gallbladder and biliary disease hospitalizations, acute pancreatitis hospitalizations, myocardial infarction (MI) or stroke hospitalizations, thyroid cancer (defined as a primary diagnosis on an inpatient claim or two diagnoses in any position in any setting at least 2 weeks apart), acute liver injury hospitalizations, psychiatric hospitalizations, suicidal ideation or self‐harm, and all‐cause mortality [9, 11, 12, 13, 14, 15, 16, 17, 18, 19, 20, 21]. All outcomes required a primary diagnosis code on an inpatient (facility) claim, with the exception of thyroid cancer, suicidal ideation or self‐harm, and all‐cause mortality. Suicidal ideation or self‐harm was defined as a diagnosis code for self‐harm, suicidal ideation, or suicide attempt in any position on an inpatient or outpatient claim. Code lists are available in the pre‐registered study protocol [23].

### 2.7 Covariates

The weighted analyses adjusted for 82 clinical and demographic covariates (Table S2a–c). Covariate status was assessed on or before the index date. Diabetes severity was defined using the adapted Diabetes Complications Severity Index (aDCSI) [25, 26]. Measures of healthcare utilization included separate counts for the following types of encounters: hospitalizations, emergency department visits, and outpatient visits during 6 months on or before the index date. A listing of all covariates is available in Table S2a–c, and code lists are available in the pre‐registered study protocol [23]. Hemoglobin A1c (HbA1c) laboratory data were available for a subset of patients. We performed a sensitivity analysis in this subset by adding HbA1c operationalized as the most recent HbA1c value within 120 days on or before the index date to the propensity score model and re‐running the weighted analysis comparing GLP‐1‐RAs to SGLT2‐Is.

### 2.8 Statistical Analyses

To address imbalances between the treatment groups in risk factors for the study outcomes, we employed propensity score‐based inverse probability weighting (IPW). Propensity scores estimated the probability of receiving a GLP‐1‐RA as opposed to the comparator of interest and were calculated using logistic regression models including all covariates. Distributions of propensity scores in each treatment group were compared visually to assess the extent of non‐overlap [24]. Stabilized inverse probability weights were calculated [27], and the weights were then applied to the data to balance covariates [27].

Analyses assessed descriptive characteristics for each comparison, Kaplan–Meier curves to estimate risk and number needed to treat or harm [NNT/H] at 6months of treatment duration, and Poisson regression models to estimate rate differences and rate ratios and their corresponding 95% confidence intervals. The weighted Poisson regression model for each analysis was run without covariates since comparability was achieved by the application of weights. Results from the Poisson models were corroborated through manually programmed calculation of weighted rates, rate ratios, and 95% confidence intervals. Absolute standardized differences (ASD) were used to assess covariate balance between treatment groups [28, 29]. Data management and analyses were conducted using Instant Health Data (IHD; Panalgo, Boston, MA) and Statistical Analysis System (SAS Institute Inc., Cary, NC, USA) platforms.

## 3 Results

### 3.1 Type 2 Diabetes Population

A total of 594,961 patients were included in the analysis: 330,684 GLP‐1‐RA users and 264,277GLT2‐I users (Table S1). The most common GLP‐1‐RA was injectable semaglutide (*n* = 140 796; 43%) followed by dulaglutide (*n* = 96 138; 29%; Table S2a). The most common SGLT2‐I was empagliflozin (*n* = 166 113; 63%) followed by dapagliflozin (*n* = 81 588; 31%; Table S2b). Discontinuation was similar between GLP‐1‐RA and SGLT2‐I users (36% and 35%, respectively). Median time to discontinuation was 8 months for GLP‐1‐RA users and 10 months for SGLT2‐I users. Before weighting, GLP‐1‐RA users were slightly younger on average, had less severe diabetes on average, were more likely than SGLT2‐I patients to be obese or female, and less likely to have had a prior MI or stroke or have a cardiologist as their index prescriber (Table S3a). After weighting, these factors were well‐balanced between treatment groups (Table 1; Table S3a).

**TABLE 1:** Covariate balance for select covariates after weighting.

	T2DM population			CWM population					
	SGLT2‐I comparison			NalBup comparison			PhenTop comparison		
	GLP‐1‐RA	SGLT2‐I	ASD	GLP‐1‐RA	NalBup	ASD	GLP‐1‐RA	PhenTop	ASD
Patients included in analysis (n)	330 684	264 277		25 296	5019		25 489	3841	—
Demographics									
Age at initiation of study drug (mean)	56.1	56.1	0.00	45.3	45.2	0.01	45.3	45.4	0.00
Sex (Female, %)	47.3	47.3	0.00	77.0	76.7	0.01	76.7	76.8	0.00
Race/ethnicity (%)									
White, not Hispanic or Latino	59.1	59.4	0.00	70.8	70.9	0.00	70.0	70.2	0.00
Black or African American, not Hispanic or Latino	10.7	10.7	0.00	8.7	8.7	0.00	9.0	8.8	0.01
Hispanic or Latino, any race	10.4	10.3	0.00	7.0	7.1	0.00	7.4	7.3	0.00
Asian, not Hispanic or Latino	4.0	4.0	0.00	1.4	1.4	0.00	1.5	1.4	0.01
Other race, not Hispanic or Latino	1.6	1.6	0.00	1.2	1.1	0.01	1.2	1.2	0.01
Unknown or undisclosed	14.1	14.0	0.00	10.7	10.9	0.00	10.9	11.1	0.01
Select comorbidities (before index date, %)									
Pre‐diabetes	9.8	9.4	0.01	—	—	—	—	—	—
Type 1 diabetes	9.5	9.6	0.00	—	—	—	—	—	—
Diabetes severity^a^	1.1	1.1	0.07	—	—	—	—	—	—
0	55.3	54.5	0.02	—	—	—	—	—	—
1	13.4	13.4	0.00	—	—	—	—	—	—
2	14.6	15.7	0.03	—	—	—	—	—	—
3	6.6	6.6	0.00	—	—	—	—	—	—
4	4.5	4.5	0.00	—	—	—	—	—	—
5+	5.7	5.2	0.02	—	—	—	—	—	—
Overweight^b^	16.7	16.5	0.00	23.1	22.8	0.01	23.2	23.3	0.00
Obese^b^	65.7	65.7	0.00	96.5	96.7	0.01	96.4	96.4	0.00
Myocardial infarction or stroke	8.5	8.5	0.00	1.8	1.8	0.01	1.8	2.2	0.03
Hypertension	83.7	83.9	0.01	46.8	47.0	0.00	46.3	46.6	0.01
Dyslipidemia	85.2	85.4	0.00	51.0	50.4	0.01	51.1	51.4	0.01
ASCVD	31.4	31.5	0.00	9.9	10.1	0.01	9.7	10.6	0.03
Heart failure	11.0	11.1	0.00	1.9	2.1	0.02	1.8	2.0	0.01

Abbreviations: ASCVD, atherosclerotic cardiovascular disease; ASD, absolute standardized difference; CWM, chronic weight management; GLP‐1‐RA, glucagon‐like peptide 1 receptor agonists; MI, myocardial infarction; *n*, number of patients; NalBup, naltrexone hydrocholoride/bupropion hydrochloride; PhenTop, phentermine/topiramate extended‐release; SGLT2‐I, sodium glucose co‐transporter 2 inhibitors; T2DM, type 2 diabetes.

^a^ Adapted Diabetes Complications Severity Index (aDCSI) score.

^b^ All available data from January 2006 were used to assess baseline covariates; as a result, patients may have diagnosis codes for both overweight and obesity due to changes in their weight status over time.

Unweighted and weighted results are presented in Table S4a and Table 2, respectively. Across the 11 outcomes, rates ranged from 0.2 per 1000 person‐years for gastroparesis in the SGLT2‐I cohort to 13.7 per 1000 person‐years for MI or stroke hospitalizations in the GLP‐1‐RA cohort (Table 2). Rate ratio estimates comparing GLP‐1‐RA to SGLT2‐I users ranged from 0.76 (95% CI: 0.64–0.91) for acute liver injury hospitalizations to 1.18 (95% CI: 1.11–1.25) for gastrointestinal hospitalizations (Table 2). NNT/H ranged from 1507 for all‐cause mortality to 9,093,156 for gastroparesis hospitalizations (Table 2). Kaplan–Meier survival curves for each outcome are presented in Figure S1. The rate of thyroid cancer was similar between treatment groups (Table 2).

**TABLE 2:** Incidence rates (per 1000 person‐years) of severe gastrointestinal events, serious cardiovascular events, thyroid cancer, acute liver injury, psychiatric hospitalizations, suicidal ideation and suicide attempts, and all‐cause mortality among patients using GLP‐1‐RAs, SGLT2‐Is, or weight management medications in the HIRD, 2016–2023.^a^

Diabetes cohorts									
Outcome	GLP‐1‐RA cohort (*N* = 330 684)			SGLT2‐I cohort (*N* = 264 277)			GLP‐1‐RA vs. SGLT2‐I		
	*n*	Person‐years^b^	Rate^c^	*n*	Person‐years^b^	Rate^c^	Rate ratio (95% CI)	Rate difference (95% CI)	6‐month NNT/H^d^
GI hospitalization	2907	313627.0	9.27	2109	267747.2	7.87	1.18 (1.11, 1.25)	1.40 (0.92, 1.88)	3412
Gastroparesis hospitalization	85	315615.0	0.27	64	269135.3	0.24	1.12 (0.81, 1.55)	0.03 (−0.05, 0.11)	9 093 156
Bowel obstruction hospitalization	771	315098.9	2.45	605	268769.6	2.25	1.09 (0.98, 1.21)	0.20 (−0.05, 0.45)	198 929
Gall bladder and biliary disease hospitalization	1725	314457.0	5.48	1295	268235.7	4.83	1.14 (1.06, 1.22)	0.65 (0.28, 1.02)	16 859
Acute pancreatitis hospitalization	665	315260.6	2.11	576	268668.1	2.14	0.98 (0.88, 1.10)	−0.03 (−0.27, 0.21)	5609
MI or stroke hospitalization	4282	312279.9	13.71	3532	266474.8	13.26	1.03 (0.99, 1.08)	0.45 (−0.15, 1.05)	3099
Thyroid cancer^e^	806	315659.9	2.55	727	269191.6	2.70	0.95 (0.86, 1.05)	−0.15 (−0.41, 0.11)	3716
Acute liver injury hospitalization	238	315550.0	0.75	266	269071.2	0.99	0.76 (0.64, 0.91)	−0.24 (−0.39, −0.09)	7259
Psychiatric hospitalization	1354	314770.2	4.30	1204	268354.6	4.49	0.96 (0.89, 1.04)	−0.19 (−0.53, 0.15)	3997
Suicidal ideation or self‐harm	782	315096.0	2.48	692	268675.2	2.57	0.96 (0.87, 1.07)	−0.09 (−0.35, 0.17)	8158
All‐cause mortality	2470	315659.9	7.83	1973	269191.6	7.33	1.07 (1.01, 1.13)	0.50 (0.05, 0.95)	1507

Chronic weight management cohorts									
Outcome	GLP‐1‐RA cohort (*N* = 25 296)			NalBup cohort (*N* = 5019)			GLP‐1‐RA vs. NalBup		
	*n*	Person‐years^b^	Rate^c^	*n*	Person‐years^b^	Rate^c^	Rate ratio (95% CI)	Rate difference (95% CI)	6‐month NNT/H^d^
GI hospitalization	105	17526.8	6.00	25	4179.1	6.06	0.99 (0.64, 1.53)	−0.06 (−2.68, 2.56)	1554
Gastroparesis hospitalization	2	17571.2	0.11	0	4191.7	0	—	0.11 (−0.05, 0.27)	11 233
Bowel obstruction Hospitalization	29	17558.7	1.63	4	4187.4	0.87	1.88 (0.63, 5.60)	0.76 (−0.31, 1.83)	4989
Gall bladder and biliary disease hospitalization	83	17538.0	4.71	6	4185.3	1.42	3.32 (1.44, 7.64)	3.29 (1.77, 4.81)	834
Acute pancreatitis hospitalization	22	17560.4	1.23	1	4191.6	0.23	5.37 (0.70, 41.35)	1.00 (0.30, 1.7)	2209
MI or stroke hospitalization	36	17553.7	2.04	8.9	4176.9	2.12	0.96 (0.46, 2.00)	−0.08 (−1.63, 1.47)	2248
Thyroid cancer^e^	86	17571.5	4.92	13.1	4191.7	3.12	1.57 (0.88, 2.82)	1.8 (−0.18, 3.78)	1640.1
Acute liver injury hospitalization	6	17570.3	0.32	0	4191.7	0	—	0.32 (0.06, 0.58)	12 558
Psychiatric hospitalization	47	17549.5	2.68	11.5	4183.3	2.74	0.98 (0.51, 1.86)	−0.06 (−1.82, 1.70)	3728
Suicidal ideation or self‐harm	37	17553.4	2.12	16.9	4177.5	4.04	0.52 (0.30, 0.93)	−1.92 (−3.97, 0.13)	1102
All‐cause mortality	11	17571.5	0.60	1.7	4191.7	0.40	1.51 (0.29, 7.73)	0.20 (−0.51, 0.91)	5396

Outcome	GLP‐1‐RA cohort (*N* = 25 489)			PhenTop cohort (*N* = 3841)			GLP‐1‐RA vs. PhenTop		
	*n*	Person‐years^b^	Rate^c^	*n*	Person‐years^b^	Rate^c^	Rate ratio (95% CI)	Rate difference (95% CI)	6‐month NNT/H^d^
GI hospitalization	107	17678.7	6.10	19	2397.6	8.08	0.75 (0.46, 1.21)	−1.98 (−5.76, 1.8)	599
Gastroparesis hospitalization	2	17722.8	0.11	1	2401.8	0.27	0.43 (0.03, 7.09)	−0.16 (−0.81, 0.49)	4260
Bowel obstruction hospitalization	29	17710.5	1.61	2	2400.6	0.91	1.77 (0.45, 6.99)	0.7 (−0.65, 2.05)	4273
Gall bladder and biliary disease hospitalization	84	17688.5	4.76	5	2400.4	2.20	2.17 (0.90, 5.22)	2.56 (0.42, 4.7)	2858
Acute pancreatitis hospitalization	22	17712.1	1.23	1	2400.5	0.49	2.51 (0.39, 15.98)	0.74 (−0.29, 1.77)	1506
MI or stroke hospitalization	37	17703.9	2.09	6	2400.2	2.28	0.92 (0.37, 2.25)	−0.19 (−2.22, 1.84)	998
Thyroid cancer^e^	90	17723.2	5.08	8	2401.9	3.35	1.52 (0.74, 3.12)	1.73 (−0.82, 4.28)	1610
Acute liver injury hospitalization	6	17721.9	0.32	0	2401.9	0	—	0.32 (0.06, 0.58)	12 450
Psychiatric hospitalization	47	17701.0	2.68	5	2399.1	2.26	1.19 (0.49, 2.89)	0.42 (−1.63, 2.47)	4970
Suicidal ideation or self‐harm	37	17705.2	2.10	4	2401.3	1.73	1.21 (0.44, 3.34)	0.37 (−1.43, 2.17)	1303
All‐cause mortality	10	17723.2	0.59	0	2401.9	0	—	0.59 (0.23, 0.95)	4088

Abbreviations: CI, confidence interval; GI, gastrointestinal; GLP‐1‐RA, glucagon‐like peptide 1 receptor agonists; MI, myocardial infarction; *n*, number of patients who experienced the outcome during follow‐up; NalBup, naltrexone hydrochloride/bupropion hydrochloride; NNT/H, number needed to treat to see benefit or harm; PhenTop, phentermine/topiramate extended‐release; SGLT2‐I, sodium glucose co‐transporter 2 inhibitors; vs., versus.

^a^ The weighted analysis adjusted for 82 clinical and demographic covariates. With the exception of cohort counts (e.g., *N* = 330 684), all values appearing in this table are weighted; as a result, patient counts for outcomes may appear as non‐whole numbers due to weighting.

^b^ Patients were followed until pregnancy, death, disenrollment, malignancy, bariatric surgery, discontinuation of their index medication, initiation of a GLP‐1‐RA (SGLT2‐I, NalBup, and PhenTop users only), initiation of a SGLT2‐I, NalBup, or PhenTop (GLP‐1‐RA users in respective comparison only), occurrence of the outcome of interest, or end of available data (12/31/2023). In the diabetes analyses, the study period began on January 1, 2016. In the weight loss analyses, the study period began on January 1, 2020.

^c^ Rate per 1000 person‐years.

^d^ NNT/Hs were calculated using the Kaplan–Meier‐based approach described by Suissa (2015; PMID 26241223). The 6‐month time point was selected since mean and median follow‐up time were between 6 and 12 months.

^e^ Two outpatient visits at least 30 days apart or one inpatient visit with a primary diagnosis code for thyroid cancer.

Findings from the sensitivity analysis in the subset of patients with available HbA1c laboratory data are presented in Table S4a. A total of 191,742 patients (105 882 GLP‐1‐RA users and 85 860 SGLT2‐I users) had HbA1c data available and were included in the sensitivity analysis. Before weighting, HbA1c was slightly imbalanced between the two treatment groups (mean of 8.3% among GLP‐1‐RA users and 8.6% among SGLT2‐I users, ASD = 0.17). After weighting, HbA1c was balanced at 8.5% in each group (ASD = 0.015). After controlling for HbA1c, rate ratios and their 95% confidence intervals did not show meaningful changes, with the HbA1c‐adjusted confidence intervals showing slightly less precision due to the smaller cohort sizes. Rate differences remained small, occasionally changing direction though not substantially in line with rate ratios (e.g., gastroparesis hospitalizations rate ratio 1.12 and 0.99 and rate difference 0.03 and −0.01 for main and sensitivity analyses, respectively). The 6‐month risk differences remained small and were similar between analyses when rounded but occasionally produced large changes in 6‐month NNT (e.g., gastroparesis hospitalizations NNT 9093156 and 32 529 for main and sensitivity analyses, respectively).

### 3.2 Chronic Weight Management Population

#### 3.2.1 GLP‐1‐RA Versus Naltrexone/Bupropion (Nal/Bup)

A total of 30,315 patients were included in the analysis: 25,296 GLP‐1‐RA users and 5019 NalBup users (Table S1). The most common GLP‐1‐RA in the GLP‐1‐RA cohort was injectable semaglutide (*n* = 16 612; 66%) followed by tirzepatide (*n* = 5503; 22%; Table S2a). Discontinuation was similar between GLP‐1‐RA and NalBup users (40% and 39%, respectively). Median time to discontinuation was 6 months for GLP‐1‐RA users and 5 months for NalBup users. Before weighting, GLP‐1‐RA users were less likely to be female, more likely to be located in the Northeast, and more likely to have dyslipidemia or have a cardiologist as their index prescriber than NalBup patients (Table S3b). After weighting, these factors were well‐balanced between treatment groups (Table 1; Table S3b).

Unweighted and weighted results are presented in Table S4b and Table 2, respectively. Across the 11 outcomes, rates ranged from 0 per 1000 person‐years for gastroparesis and acute liver injury hospitalization outcomes in the NalBup cohort to 6.1 per 1000 person‐years for gastrointestinal hospitalizations in the NalBup cohort (Table 2). Rate ratio estimates comparing GLP‐1‐RA to NalBup users ranged from 0.52 (95% CI: 0.30–0.93) for suicidal ideation or self‐harm to 5.37 (95% CI: 0.70–41.35) for acute pancreatitis hospitalizations (Table 2). NNT/H ranged from 834 for biliary disease hospitalizations to 12,558 for acute liver injury hospitalizations (Table 2). Kaplan–Meier survival curves for each outcome are presented in Figure S2.

#### 3.2.2 GLP‐1‐RA Versus Phentermine/Topiramate (Phen/Top)

A total of 29,330 patients were included in the analysis: 25,489 GLP‐1‐RA users and 3841 PhenTop users (Table S1). The most common GLP‐1‐RA was injectable semaglutide (*n* = 16 729; 66%) followed by tirzepatide (*n* = 5550; 22%; Table S2a). Discontinuation was less frequent in GLP‐1‐RA than in PhenTop users (40% and 48%, respectively). Median time to discontinuation in patients censored for discontinuation was 6 months and 5 months, respectively. Before weighting, GLP‐1‐RA users were less likely to be female, less likely to be located in the Midwest, more likely to be located in the Northeast, and more likely to have hypertension, dyslipidemia, or atherosclerotic cardiovascular disease, or have a cardiologist prescribe their index medication than PhenTop patients (Table S3c). After weighting, these factors were well‐balanced between treatment groups (Table 1; Table S3c).

**FIGURE 1:**
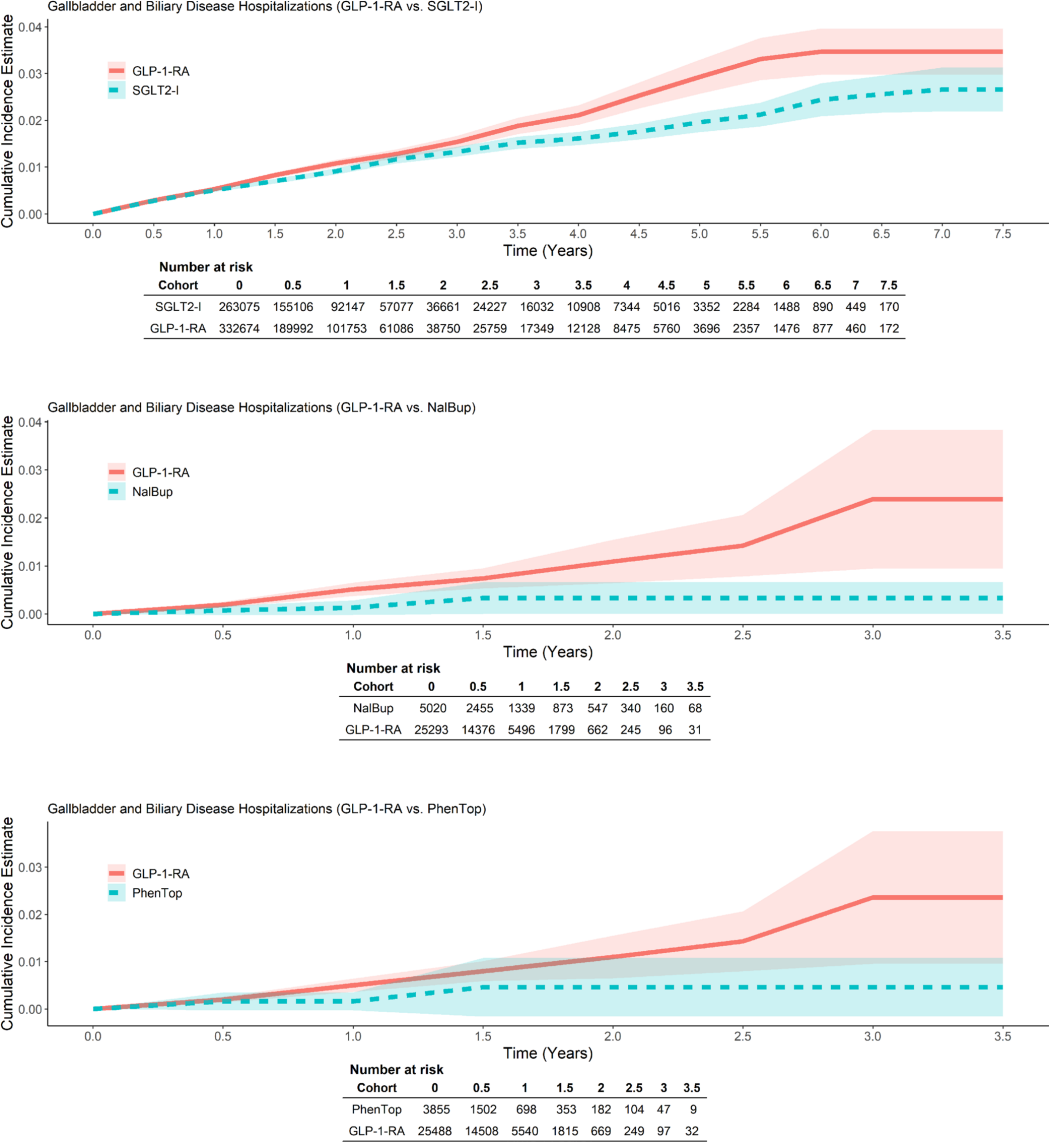
Adjusted (weighted) Kaplan–Meier cumulative incidence curves for gallbladder and biliary disease hospitalization.

Unweighted and weighted results are presented in Table S4c and Table 2, respectively. Across the 11 outcomes, rates ranged from 0 per 1000 person‐years for acute liver injury hospitalizations and all‐cause mortality in the PhenTop cohort to 8.1 per 1000 person‐years for gastrointestinal hospitalizations in the PhenTop cohort (Table 2). Rate ratio estimates comparing GLP‐1‐RA to PhenTop users ranged from 0.43 (95% CI: 0.03–7.09) for gastroparesis hospitalizations to 2.51 (95% CI: 0.39–15.98) for acute pancreatitis hospitalizations (Table 2). NNT/H ranged from 599 for gastrointestinal hospitalizations to 12 450 for acute liver injury hospitalizations (Table 2). Kaplan–Meier survival curves for each outcome are presented in Figure S3. The rate of gallbladder and biliary disease hospitalizations was higher in the GLP‐1‐RA cohort than in the PhenTop cohort, with similar time‐to‐occurrence between groups until approximately 6 months (0.5 years) of treatment (Table 2; Figure 1).

### 3.3 Results Most Consistent Across Indications

The incidence of bowel obstruction hospitalizations was slightly higher in the GLP‐1‐RA cohorts in the T2DM and CWM populations (GLP‐1‐RA vs. SGLT2‐I RR: 1.09; 95% CI: 0.98–1.21; GLP‐1‐RA vs. NalBup RR: 1.88; 95% CI: 0.63–5.60; and GLP‐1‐RA vs. PhenTop RR: 1.77; 95% CI: 0.45–6.99; Table 2), though estimates were imprecise due to a low number of events.

The incidence of gallbladder and biliary disease hospitalizations was slightly higher in the GLP‐1‐RA cohort in the T2DM population (GLP‐1‐RA vs. SGLT2‐I RR: 1.14; 95% CI: 1.06–1.22), and more strongly associated in the CWM population (GLP‐1‐RA vs. NalBup RR: 3.32; 95% CI: 1.44–7.64; and GLP‐1‐RA vs. PhenTop RR: 2.17; 95% CI: 0.90–5.22; Table 2). Kaplan–Meier time‐to‐event curves showed largest separation after between approximately2 and 33 years of treatment (Figure 1).

**FIGURE 2:**
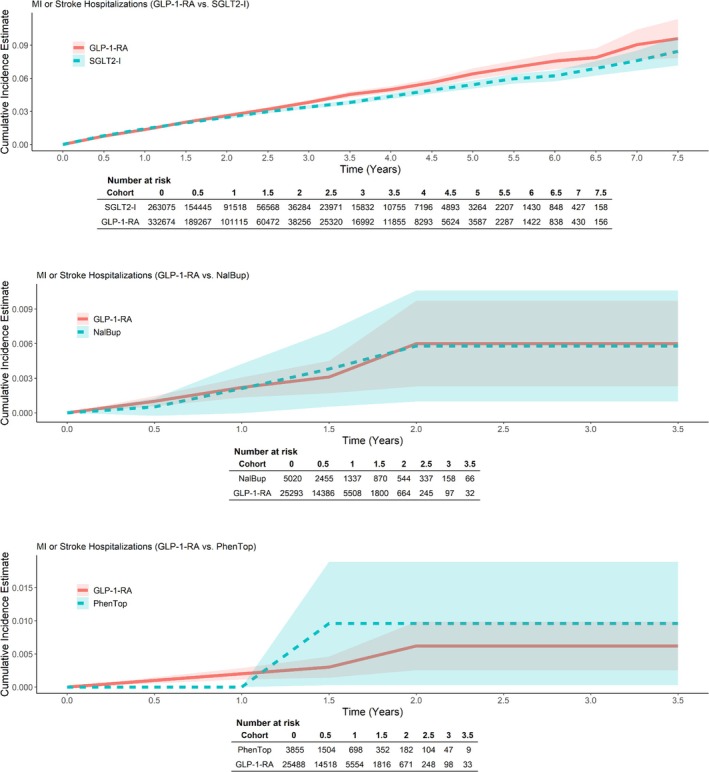
Adjusted (weighted) Kaplan–Meier cumulative incidence curves for myocardial infarction or stroke hospitalization.

The incidence of myocardial infarction and stroke hospitalizations was similar across treatment groups in both T2DM and CWM populations. Kaplan–Meier time‐to‐event curves were similar between treatment groups but imprecise for the CWM population (Figure 2).

## 4 Discussion

This study assessed the impact of contemporary GLP‐1‐RA therapies on several serious clinical outcomes in a real‐world setting. GLP‐1‐RAs were associated with a higher incidence of hospitalizations for gall bladder and biliary disease in both T2DM and CWM indications, corroborating results from clinical trials [17, 30]. Additionally, Kaplan–Meier curves showed increasing separation after approximately2 to 33 years of treatment, consistent with the greater risk for gall bladder and biliary disease associated with longer‐term use seen in the trials [30].

In contrast to clinical trials, rates of myocardial infarction and stroke hospitalizations were similar across treatment groups, although results were somewhat imprecise in the CWM population. Cardiovascular events comprising MI and stroke were the most commonly observed outcomes among patients with T2DM. Unlike prior cardiovascular outcomes trials with placebo comparators in patients with pre‐existing cardiovascular disease [17, 18, 19], this study found no reduction in cardiovascular events with GLP‐1‐RA use. This lack of apparent benefit of GLP‐1‐RAs on cardiovascular outcomes could be due partly to differences in design. For T2DM patients, we compared GLP‐1‐RAs to SGLT2‐Is, which also have been associated with a reduced risk of MI and stroke in multiple studies [31]. In addition, trials studied patients with a history of cardiovascular disease, which were likely higher risk populations than included here. For CWM patients, we compared GLP‐1‐RAs to medications that have not been reported to reduce the risk of MI or stroke but still observed similar rates of cardiovascular events. Less than 2% of individuals in the CWM population had a history of MI or stroke, making our CWM study population a lower‐risk population than was examined in cardiovascular outcomes trials. The absence of a reduction in the risk of MI and stroke in the CWM cohorts may suggest that the cardiovascular benefits of GLP‐1‐RAs are concentrated among high‐risk patients included in trials [17, 18]. There is currently little evidence for a cardiovascular benefit with GLP‐1‐RAs among patients without a history of MI or stroke.

The rate of acute liver injury hospitalizations was lower in GLP‐1‐RA users in the T2DM population. However, these results require further research because although GLP‐1‐RAs have shown beneficial effects in chronic hepatic conditions such as metabolic dysfunction‐associated steatohepatitis, their beneficial effect (or the detrimental effect of SGLT2‐Is) on acute hepatic conditions has not been previously observed [15, 20, 21, 32].

We observed no increased risk of suicidal ideation among GLP‐1‐RA users in the T2DM population, consistent with other real‐world study results [33]. The lower rate of suicidal ideation or self‐harm in GLP‐1‐RA users compared with NalBup users in the CWM population (RR: 0.52; 95% CI: 0.30–0.93) could be due to an increased risk in the NalBup comparator group as suggested by a warning on the FDA‐approved package insert for bupropion [34, 35]. Wang et al. also observed a lower risk of incident suicidal ideation in GLP‐1‐RA users compared with users of non‐GLP‐1‐RA anti‐obesity medications including bupropion (HR = 0.27; 95% CI = 0.20–0.36) [36]. The evidence suggests no increased risk of suicidal ideation with GLP‐1‐RA use relative to the other therapeutic options studied.

**FIGURE 3:**
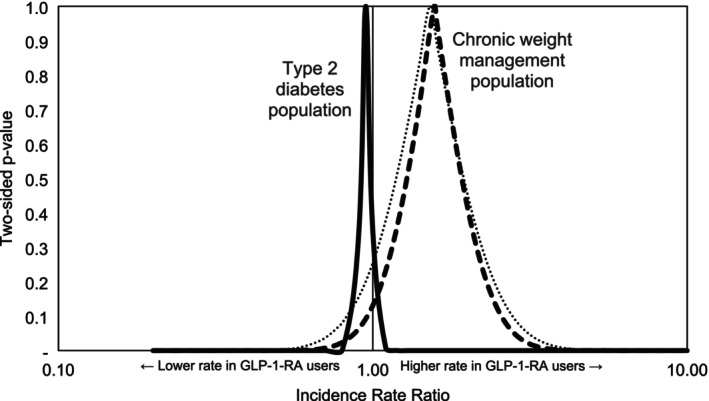
*p*‐value functions of rate ratios for thyroid cancer. GLP‐1‐RA, glucagon‐like peptide 1 receptor agonists; NalBup, naltrexone hydrocholoride/bupropion hydrochloride; PhenTop, phentermine/topiramate extended‐release; SGLT2‐I, sodium glucose co‐transporter 2 inhibitors. Solid line: Comparing GLP‐1‐RA to SGLT2‐I; Dotted line: Comparing GLP‐1‐RA to PhenTop; Dashed line: Comparing GLP‐1‐RA to NalBup. This plot displays all *p*‐values for a range of possible rate ratios. The probabilities shown are for the observed data given each hypothesized RR. The axis values of this plot can be used to infer confidence limits at varying levels of confidence. For example, X‐axis values at the 0.05 level of the Y‐axis represent the 95% confidence interval limits. Similarly, the X‐axis values at the 0.10 level of the Y‐axis represent the 90% confidence interval limits. The peak of each curve represents the study point estimate (rate ratio). The two‐sided null hypothesis *p*‐value (familiar for its common use in statistical significance testing) is the Y‐axis value at which the X‐axis value is 1 (e.g., two‐sided *p* ~ 0.1 when comparing GLP‐1‐RA to NalBup).

This study found no increased rate of thyroid cancer in T2DM patients taking GLP‐1‐RAs versus SGLT2‐Is, but an imprecise increased rate in CWM patients taking GLP‐1‐RAs versus two different comparator weight management drugs (Figure 3). The FDA warning for thyroid cancer on the package insert of GLP‐1‐RA is based on preclinical studies in rodents and is focused on C‐cell tumors, including medullary thyroid carcinoma [37]. Several other studies, mainly in T2DM patients, present imprecise results for thyroid cancer consistent with no increased risk as well as with a small increase in risk [38, 39, 40, 41]. The higher rates of thyroid cancer in the CWM population overall and in CWM patients taking GLP‐1‐RAs versus other CWM medications may reflect the higher incidence of thyroid/medullary thyroid cancer in females in their late 40s/early 50s, consistent with the differences in the demographics observed in our T2DM and CWM study populations, as well as more aggressive thyroid cancer screening in patients on GLP‐1 s [42, 43].

Our all‐cause mortality results suggested a possible increased risk in those exposed to GLP‐1‐RAs in both the T2DM and CWM populations, but these results raise questions. First, the association was weak, nonspecific as to the cause of death, and mortality was assessed from various sources of uncertain accuracy. In addition, in two large cardiovascular outcomes trials for semaglutide (a GLP‐1‐RA) [17, 18], all‐cause mortality rates were not different compared to placebo in individuals with T2DM, while there was a 19% reduction observed in those with overweight/obesity and pre‐existing cardiovascular disease. In contrast, there was a 32% reduction in the risk of all‐cause mortality in patients with pre‐existing cardiovascular disease taking empagliflozin (an SGLT2‐I) vs. placebo [44].

The current study has several strengths. This study employed an active comparator, new user study design that eliminates certain selection biases that can cloud interpretation of real‐world studies [45]. The study also assessed serious clinical outcomes of interest to clinicians and patients in the two main populations of interest. The large cohort sizes drawn from a geographically diverse U.S. population provide relatively precise and generalizable results. In addition, our study did not restrict the population to patients who did not previously experience the outcomes under study. The reason is that people who have a demonstrated susceptibility may be at greatest risk of a potential medication effect. Thus, in analyzing the occurrence of acute cardiovascular outcomes such as MI, we did not exclude patients with a previous MI. Instead, we used propensity score weighting to create balance between the treatment groups in the percentage of patients with prior experience of the study outcomes. To avoid counting as cases a diagnosis that referred to a history of the outcome (e.g., a history of MI), we required a primary hospital diagnosis for all outcomes except suicidal ideation and thyroid cancer, which were identified from both inpatient and outpatient diagnoses. Finally, whereas prior real‐world research has focused on older GLP‐1‐RA therapies, this study has broad representation of the full spectrum of GLP‐1‐RA therapies in the United States, including over 20,000 patients each on oral semaglutide and tirzepatide.

This study also has several limitations. The median follow‐up time in this study was about 1 year, versus the more than 2 and 3 years in the SUSTAIN‐6 and SELECT trials, respectively [17, 18]. It is possible that longer durations of use may be associated with different results, and that discontinuation in real‐world use contributes to different results from clinical trials. Among the approximately 40% of GLP‐1‐RA users who discontinued GLP‐1‐RA use during the study period, the median time to discontinuation was 8 months in the T2DM cohort and 6 months in the CWM cohorts, consistent with other real‐world data studies [46, 47]. Our follow‐up period was restricted to active treatment. While the gastrointestinal, cardiovascular, and psychiatric outcomes we examined could be expected to manifest during active treatment, thyroid cancer could relate to a prior, separate exposure, and any difference seen could be due to an “acceleration” of the cancer's development in relation to one drug versus another or to increased surveillance in GLP‐1‐RA users. We observed a null effect for GLP‐1‐RA versus SGLT2‐I in the T2DM population during active treatment. This observation is consistent with prior literature that looked beyond active treatment [39, 41], including a Scandinavian cohort study with an average follow‐up period of 3.9 years among GLP‐1‐RA users in the main analysis [41], and a Korean population‐based cohort study that included a one‐year lag period and an average of 2.9 years of follow‐up [39]. In contrast, we observed an imprecise, though elevated incidence of thyroid cancer diagnoses in GLP‐1‐RAs relative to NalBup or PhenTop in the CWM population during active treatment with an average follow‐up of less than 1 year. Further studies could expand follow‐up to include post‐discontinuation follow‐up time and explore differences in thyroid cancer screening as well as past exposures that are known carcinogens that may be associated with later GLP‐1‐RA use (as opposed to NalBup or PhenTop). An additional limitation of this study is that only one fill of a study drug was required to be included in a particular cohort. It is possible that patients may fill a prescription but not use it. If this happened, we would misclassify unexposed patients as exposed, and any effect of medications would be attenuated. Future analyses could examine findings among patients with at least two fills who are more likely to have actually used a dispensed medication. Finally, this study relied on administrative health care data, which can be affected by biases that are difficult to control. Further studies could consider including one or more negative control outcomes to help evaluate validity.

This study identified serious clinical events using ICD‐10‐CM coding from administrative claims, which could be subject to erroneous coding or errors of omission. This limitation was addressed by using primary hospital discharge diagnoses where appropriate. Primary hospital discharge diagnoses drive billing and receive greater scrutiny than outpatient diagnoses unrelated to payments. They also represent more serious conditions that are thought to be more accurately ascertained. When outpatient diagnoses were permitted for thyroid cancer, we required multiple diagnoses at least 2 weeks apart. Of note, ICD‐10‐CM coding for thyroid cancer does not allow for differentiation by thyroid cancer type. Suicidal ideation or self‐harm outcomes required a diagnosis code for self‐harm, suicidal ideation, or suicide attempt in any position on an inpatient or outpatient claim. Mortality data lacked cause of death information, and while the sensitivity of the integrated death data sources is high (above 80%) [48], there still may be missed cases. Finally, pharmacy claims indicate medications dispensed but do not ensure that individuals used the medication as prescribed.

## 5 Conclusions

The real‐world evidence generated in the current study demonstrates that the serious clinical events examined are uncommon, and large populations are needed to measure these effects with precision. Nevertheless, these analyses point to important similarities and differences in rates between GLP‐1‐RAs and available therapeutic options in individuals using these medications for T2DM or CWM. Evidence suggests increased rates of hospitalizations for gall bladder and biliary disease for GLP‐1‐RAs relative to certain CWM and T2DM therapies. Additionally, we did not observe a cardiovascular benefit, and persistence with GLP‐1‐RAs and comparator therapies was suboptimal across both indications and could be a barrier to realizing certain benefits.

### 5.1 Plain Language Summary

In this study, we evaluated several safety outcomes that have been reported to be associated with glucagon‐like peptide‐1 receptor agonists (GLP‐1‐RAs) versus other drugs prescribed for type 2 diabetes (T2DM) or weight management. Health insurance data from the United States were used to identify large populations using these drugs in real‐world clinical care. We studied almost 600,000 people using these drugs for T2DM and over 30,000 using them for weight management. Discontinuation of study drugs was common, with about 40% stopping therapy within a year. Hospitalizations due to gall bladder and biliary conditions were more common among people using GLP‐1‐RAs. Heart attack and stroke rates were similar among the treatment groups. There was no increase in suicide or thoughts of suicide in people taking GLP‐1‐RAs, and people with T2DM taking GLP‐1‐RAs had fewer severe liver problems. This study supplements earlier research looking at the safety of GLP‐1‐RAs and informs individuals and their healthcare providers about the safety of these drugs.

## Ethics Statement

The management of all data and study materials conformed with the Health Insurance Portability and Accountability Act (HIPAA). A limited dataset, which excluded patient‐identifying information, was used for all analyses, as defined by the HIPAA Privacy Rule; therefore, Institutional Review Board approval was not required.

## Conflicts of Interest

This work was funded by CarelonRx, a pharmacy benefit manager. This study was designed and led by Carelon Research, a healthcare research company that frequently does contract research for pharmaceutical companies, including companies that manufacture or market the medications in this study. Both CarelonRx and Carelon Research are part of Elevance Health, a health insurance provider. The authors have declare no conflicts of interest.

## Supporting information


**Data S1:** Supporting Information.
