# Supplementary material for: Comparative Safety of Glucagon‐Like Peptide 1 Receptor Agonists (GLP‐1‐RAs) in Type 2 Diabetes and Chronic Weight Management: A Real‐World Data Study
Source: Pharmacoepidemiol Drug Saf. 2025 Sep 14;34(9):e70214. doi: 10.1002/pds.70214 (PMC12433752; doi:10.1002/pds.70214)

**Appendices**

**Table of Contents**

[Table S1. Cohort assembly for all analyses. 2](#_Toc196921726)

[Table S2a. GLP-1-RA index medications.^1^ 4](#_Toc196921727)

[Table S2b. SGLT2-I index medications.^1^ 4](#_Toc196921728)

[Table S3a. Covariate balance for GLP-1-RAs vs. SGLT2-Is among patients with type 2 diabetes, 2016-2023. 5](#_Toc196921729)

[Table S3b. Covariate balance for GLP-1-RAs vs. NalBup among non-diabetic patients for chronic weight management, 9](#_Toc196921730)

[2020-2023. 9](#_Toc196921731)

[Table S3c. Covariate balance for GLP vs. PhenTop among non-diabetic patients for chronic weight management, 2020-2023. 12](#_Toc196921732)

[Table S4a. Incidence, rate ratio, and NNT/H estimates for study outcomes for GLP-1-RAs vs. SGLT2-I among patients with type 2 diabetes, 2016-2023. 15](#_Toc196921733)

[Table S4b. Incidence, rate ratio, and NNT/H estimates for study outcomes for GLP-1-RAs vs. NalBup among patients without prediabetes or diabetes for chronic weight management, 2020-2023. 17](#_Toc196921734)

[Table S4c. Incidence, rate ratio, and NNT/H estimates for study outcomes for GLP-1-RAs vs. PhenTop among patients without prediabetes or diabetes for chronic weight management, 2020-2023. 18](#_Toc196921735)

[Figure S1. Weighted Kaplan-Meier Plots – Type 2 Diabetes Analysis. 19](#_Toc196921736)

[Figure S2. Weighted Kaplan-Meier Plots – Chronic Weight Management Analyses with NalBup as comparator. 21](#_Toc196921737)

[Figure S3. Weighted Kaplan-Meier Plots – Chronic Weight Management Analyses with PhenTop as comparator. 23](#_Toc196921738)

# Table S1. Cohort assembly for all analyses.

| **Study entry criteria: Type 2 diabetes analysis (SGLT2-I as comparator)** | **GLP-1-RA cohort** | **SGLT2-I cohort** | **Total** |
| --- | --- | --- | --- |
| Claim for study drug (GLP-1-RA or SGLT2-I) during intake period (01/01/2016-12/31/2023) | 869,799 | 650,454 | 1,520,253 |
| Age at least 18+ years on index date | 868,602 | 650,260 | 1,518,862 |
| At least 6 months of continuous enrollment before index date | 612,274 | 442,943 | 1,055,217 |
| Type 2 diabetes diagnosis on or before index date | 512,635 | 410,280 | 922,915 |
| No SGLT2-I claim before index date^1^ | 367,767 | 361,056 | 728,823 |
| No GLP-1-RA claim before index date^2^ or death date before index date | 330,743 | 264,317 | 595,060 |
| No missing covariate data or a death date occurring before the index date^3^ | 330,698 | 264,294 | 594,992 |
| Index date not on last day of study period^4^ | 330,684 | 264,277 | 594,961 |
|  |  |  |  |
| **Study entry criteria: Chronic weight management analysis (NalBup as comparator)** | **GLP-1-RA cohort** | **Nal Bup cohort** | **Total** |
| Claim for study drug (GLP-1-RA or NalBup) during intake period (01/01/2016-12/31/2023) | 869,799 | 34,229 | 904,028 |
| Age at least 18+ years on index date | 868,602 | 34,201 | 902,803 |
| At least 6 months of continuous enrollment before index date | 612,274 | 29,510 | 641,784 |
| Weight loss indication^5^ | 328,345 | 21,364 | 349,709 |
| No NalBup claim before index date^6^ | 324,942 | 20,471 | 345,413 |
| No GLP-1-RA claim before index date^7^ | 303,952 | 19,319 | 323,271 |
| No prediabetes, diabetes, or diabetes medications^8^ | 30,192 | 13,894 | 44,086 |
| Index year 2020+ | 28,191 | 5,027 | 33,218 |
| Commercially insured | 25,302 | 5,020 | 30,322 |
| No missing covariate data or a death date occurring before the index date^3^ | 25,297 | 5,020 | 30,317 |
| Index date not on last day of study period^4^ | 25,296 | 5,019 | 30,315 |
|  |  |  |  |
| **Study entry criteria: Chronic weight management analysis (PhenTop as comparator)** | **GLP-1-RA cohort** | **PhenTop cohort** | **Total** |
| Claim for study drug (GLP-1-RA or PhenTop) during intake period (01/01/2016-12/31/2023) | 869,799 | 13,315 | 883,114 |
| Age at least 18+ years on index date | 868,602 | 13,270 | 881,872 |
| At least 6 months of continuous enrollment before index date | 612,274 | 11,220 | 623,494 |
| Weight loss indication^5^ | 328,345 | 8,801 | 337,146 |
| No PhenTop claim before index date^9^ | 327,029 | 8,258 | 335,287 |
| No GLP-1-RA claim before index date^10^ | 305,894 | 7,690 | 313,584 |
| No prediabetes, diabetes, or diabetes medications^8^ | 30,437 | 5,265 | 35,702 |
| Index year 2020+ | 28,390 | 3,845 | 32,235 |
| Commercially insured | 25,496 | 3,842 | 29,338 |
| No missing covariate data or a death date occurring before the index date^3^ | 25,490 | 3,841 | 29,331 |
| Index date not on last day of study period^4^ | 25,489 | 3,841 | 29,330 |

**Abbreviations:** GLP-1-RA, glucagon-like peptide 1 receptor agonists; NalBup, naltrexone hydrocholoride/bupropion hydrochloride; PhenTop, phentermine/topiramate extended-release; SGLT2-I, sodium glucose co-transporter 2 inhibitors.

^1^For GLP-1-RA cohort, also excluded GLP-1-RA users with SGLT2-I fill on the index date.

^2^For SGLT2-I cohort, also excluded SGLT2-I users with GLP-1-RA fill on the index date.

^3^Excluded from propensity score model and weighted estimates.

^4^Included in propensity score model but not in rate models due to contributing no person-time to the analyses.

^5^Obesity diagnosis on or 6 months before the index date or overweight diagnosis on or 6 months before the index date and a diagnosis of type 2 diabetes, hypertension, or dyslipidemia on or before the index date.

^6^For GLP-1-RA cohort, also excluded GLP-1-RA users with NalBup fill on the index date.

^7^For NalBup cohort, also excluded NalBup users with GLP-1-RA fill on the index date.

^8^Excludes patients with diagnosis codes for type 1 or type 2 diabetes or prediabetes, or fills for metformin, DPP4s, insulin, sulfonylureas, other diabetes drugs (bromocriptine mesylate, donislecel, nateglinide, pioglitazone, pramlintide, repaglinide, rosiglitazone, teplizumab, or troglitazone), thiazolidinediones or SGLT2-Is.

^9^For GLP-1-RA cohort, also excluded GLP-1-RA users with PhenTop fill on the index date.

^10^For PhenTop cohort, also excluded NalBup users with GLP-1-RA fill on the index date.

# Table S2a. GLP-1-RA index medications.^1^

|  | **Type 2 diabetes analysis**  **comparing to SGLT2-Is** |  | **Chronic weight**  **management analysis**  **comparing to NalBup** |  | **Chronic weight**  **management analysis**  **comparing to PhenTop** |  |
| --- | --- | --- | --- | --- | --- | --- |
| **Drug^2^** | **n** | **%** | **n** | **%** | **n** | **%** |
| Albiglutide | 1,379 | 0.4% | 0 | --- | 0 | --- |
| Dulaglutide | 96,138 | 29.1% | 1,142 | 4.5% | 1,165 | 4.6% |
| Exenatide | 10,997 | 3.3% | 25 | 0.1% | 26 | 0.1% |
| Liraglutide | 32,376 | 9.8% | 653 | 2.6% | 654 | 2.6% |
| Lixisenatide | 2,438 | 0.7% | 0 | --- | 0 | --- |
| Semaglutide (inj.) | 140,796 | 42.6% | 16,612 | 65.7% | 16,729 | 65.6% |
| Semaglutide (oral) | 26,618 | 8.0% | 1,369 | 5.4% | 1,373 | 5.4% |
| Tirzepatide | 20,057 | 6.1% | 5,503 | 21.8% | 5,550 | 21.8% |
| **Combination** |  |  |  |  |  |  |
| With insulin | 3,184 | 1.0% | 0 | --- | 0 | --- |
| **Total** | **330,799** | **100%** | **25,304** | **100%** | **25,497** | **100%** |
| **Cohort N** | **330,684** | **---** | **25,296** | **---** | **25,489** | **---** |

^1^A small number (<0.05%) had more than one GLP-1-RA dispensed on their index date.

A total of n=3 patients in the diabetes analysis had missing information on GLP-1-RA type.

^2^Includes combination drugs.

# Table S2b. SGLT2-I index medications.^1^

| **Drug^2^** | **n** | **%** |
| --- | --- | --- |
| Canagliflozin | 15,250 | 5.8% |
| Dapagliflozin | 81,588 | 30.9% |
| Empagliflozin | 166,113 | 62.9% |
| Ertugliflozin | 1,328 | 0.5% |
| **Combination** |  |  |
| With DPP-4 | 4,073 | 1.5% |
| With metformin | 18,817 | 7.1% |
| **Total** | **264,381** | **100%** |
| **Cohort N** | **264,277** | **---** |

^1^A small number (<0.05%) had more than one GLP-1-RA dispensed on their index date.

A total of n=102 patients had missing information on SGLT2-I type.

^2^Includes combination drugs.

# Table S3a. Covariate balance for GLP-1-RAs vs. SGLT2-Is among patients with type 2 diabetes, 2016-2023.

|  | **Unweighted** | | | **Weighted** | | |
| --- | --- | --- | --- | --- | --- | --- |
|  | **GLP-1-RA cohort** | **SGLT2-I cohort** |  | **GLP-1-RA cohort** | **SGLT2-I cohort** |  |
| **Covariate** | **n=330,743** | **n=264,317** | **ASD** | **n=330,684** | **n=264,277** | **ASD** |
| **Demographics** |  |  |  |  |  |  |
| Age (Mean) | 54.2 | 58.2 | 0.34 | 56.1 | 56.1 | 0.00 |
| Sex (Female, %) | 54.6 | 39.0 | 0.32 | 47.3 | 47.3 | 0.00 |
| Region (%) |  |  | 0.13 |  |  | 0.04 |
| - Midwest | 25.7 | 23.7 | 0.05 | 25.0 | 24.7 | 0.01 |
| - Northeast | 12.0 | 13.0 | 0.03 | 12.3 | 12.4 | 0.00 |
| - South | 42.6 | 38.5 | 0.08 | 40.6 | 40.5 | 0.00 |
| - West | 18.4 | 22.9 | 0.11 | 20.6 | 20.7 | 0.00 |
| - Missing/Unknown | 1.3 | 1.9 | 0.05 | 1.6 | 1.6 | 0.00 |
| Payor commercially insured (%) | 85.1 | 78.7 | 0.17 | 82.0 | 82.0 | 0.00 |
| Index year 2020 or greater (%) | 73.8 | 63.8 | 0.22 | 68.0 | 68.3 | 0.01 |
| Race/Ethnicity (%) |  |  | 0.19 |  |  | 0.00 |
| - White, not Hispanic or Latino | 61.7 | 56.3 | 0.11 | 59.1 | 59.4 | 0.00 |
| - Black or African American, not Hispanic or Latino | 11.2 | 10.4 | 0.03 | 10.7 | 10.7 | 0.00 |
| - Hispanic or Latino, any race | 9.7 | 11.1 | 0.05 | 10.4 | 10.3 | 0.00 |
| - Asian, not Hispanic or Latino | 2.8 | 5.6 | 0.14 | 4.0 | 4.0 | 0.00 |
| - Other race, Not Hispanic or Latino | 1.5 | 1.6 | 0.01 | 1.6 | 1.6 | 0.00 |
| - Unknown or undisclosed | 13.2 | 15.0 | 0.05 | 14.1 | 14.0 | 0.00 |
| **Prior diagnosis of a study outcome (%)** |  |  |  |  |  |  |
| Gastrointestinal conditions | 32.2 | 30.4 | 0.04 | 31.6 | 31.6 | 0.00 |
| Gastroparesis | 1.2 | 0.0 | 0.00 | 1.2 | 1.3 | 0.00 |
| Bowel obstruction | 2.3 | 0.0 | 0.02 | 2.4 | 2.4 | 0.00 |
| Gall bladder and biliary disease | 9.3 | 0.1 | 0.01 | 9.3 | 9.3 | 0.00 |
| Acute pancreatitis | 1.4 | 0.0 | 0.07 | 2.0 | 1.9 | 0.01 |
| Myocardial infarction or stroke | 6.4 | 0.1 | 0.16 | 8.5 | 8.5 | 0.00 |
| Thyroid cancer | 0.6 | 0.0 | 0.00 | 0.6 | 0.6 | 0.00 |
| Acute liver injury | 2.4 | 0.0 | 0.03 | 2.7 | 2.7 | 0.00 |
| Psychiatric hospitalization | 1.8 | 0.0 | 0.02 | 1.7 | 1.8 | 0.00 |
| Self-harm, suicidal ideation, or suicide attempt | 1.1 | 0.0 | 0.03 | 0.9 | 1.0 | 0.00 |
| **Contraindications for any study drug (%)** |  |  |  |  |  |  |
| Multiple endocrine neoplasia (MEN) type II | 0.0 | 0.0 | 0.00 | 0.0 | 0.0 | 0.00 |
| **Diabetes status (%)** |  |  |  |  |  |  |
| Pre-diabetes | 12.8 | 6.6 | 0.21 | 9.8 | 9.4 | 0.01 |
| Type 2 diabetes | 100.0 | 100.0 | - | 100.0 | 100.0 | . |
| Type 1 diabetes | 9.2 | 9.1 | 0.00 | 9.5 | 9.6 | 0.00 |
| Diabetes severity | 0.9 | 1.2 | 0.22 | 1.1 | 1.1 | 0.07 |
| - 0 | 59.8 | 50.7 | 0.18 | 55.3 | 54.5 | 0.02 |
| - 1 | 13.5 | 13.0 | 0.01 | 13.4 | 13.4 | 0.00 |
| - 2 | 13.4 | 17.0 | 0.10 | 14.6 | 15.7 | 0.03 |
| - 3 | 5.8 | 7.3 | 0.06 | 6.6 | 6.6 | 0.00 |
| - 4 | 3.5 | 5.5 | 0.10 | 4.5 | 4.5 | 0.00 |
| - 5+ | 4.2 | 6.5 | 0.10 | 5.7 | 5.2 | 0.02 |
| **Metabolic conditions (%)** |  |  |  |  |  |  |
| Overweight | 15.2 | 18.5 | 0.09 | 16.7 | 16.5 | 0.00 |
| Obese | 73.5 | 56.5 | 0.36 | 65.7 | 65.7 | 0.00 |
| Hypertension | 81.9 | 85.4 | 0.10 | 83.7 | 83.9 | 0.01 |
| Dyslipidemia | 83.1 | 87.4 | 0.12 | 85.2 | 85.4 | 0.00 |
| **Autoimmune conditions (%)** |  |  |  |  |  |  |
| Ankylosing spondylitis | 0.4 | 0.3 | 0.01 | 0.3 | 0.3 | 0.00 |
| Bechet’s disease | 0.0 | 0.0 | 0.00 | 0.0 | 0.0 | 0.00 |
| Celiac | 0.5 | 0.4 | 0.02 | 0.4 | 0.4 | 0.00 |
| Guillain-Barre syndrome (GBS) | 0.1 | 0.1 | 0.00 | 0.1 | 0.1 | 0.00 |
| Lupus | 0.0 | 0.0 | 0.03 | 0.0 | 0.0 | 0.00 |
| Myositis | 7.5 | 6.8 | 0.03 | 7.3 | 7.2 | 0.00 |
| Psoriasis | 4.0 | 3.4 | 0.03 | 3.7 | 3.7 | 0.00 |
| Rheumatoid arthritis | 3.6 | 3.1 | 0.03 | 3.4 | 3.4 | 0.00 |
| Sjogren’s syndrome | 0.7 | 0.5 | 0.02 | 0.6 | 0.6 | 0.00 |
| Systemic sclerosis | 0.1 | 0.1 | 0.00 | 0.1 | 0.1 | 0.00 |
| **Cardiovascular disease (%)** |  |  |  |  |  |  |
| Atherosclerotic cardiovascular disease (ASCVD) | 27.0 | 36.0 | 0.19 | 31.4 | 31.5 | 0.00 |
| Heart failure | 8.1 | 14.6 | 0.21 | 11.0 | 11.1 | 0.00 |
| **Genetic disorders (%)** |  |  |  |  |  |  |
| Ehlers-Danlos syndrome | 0.1 | 0.0 | 0.02 | 0.1 | 0.1 | 0.00 |
| Myotonic or muscular dystrophy | 0.1 | 0.1 | 0.00 | 0.1 | 0.1 | 0.00 |
| **Gastrointestinal disease (%)** |  |  |  |  |  |  |
| Gastro-esophageal reflux disease | 36.8 | 33.4 | 0.07 | 35.4 | 35.4 | 0.00 |
| Inflammatory bowel disease | 10.3 | 9.6 | 0.02 | 10.1 | 10.2 | 0.00 |
| Irritable bowel syndrome | 5.5 | 4.2 | 0.06 | 5.0 | 5.0 | 0.00 |
| **Neurological disease (%)** |  |  |  |  |  |  |
| Amyotrophic lateral sclerosis | 0.0 | 0.0 | 0.00 | 0.0 | 0.0 | 0.00 |
| Dysautonomia | 1.7 | 1.6 | 0.00 | 1.7 | 1.7 | 0.00 |
| Migraine | 10.4 | 6.3 | 0.15 | 8.6 | 8.6 | 0.00 |
| Multiple sclerosis | 0.5 | 0.4 | 0.02 | 0.5 | 0.5 | 0.00 |
| Parkinson’s | 0.3 | 0.4 | 0.02 | 0.4 | 0.4 | 0.00 |
| Sleep disorder | 39.9 | 31.4 | 0.18 | 36.0 | 36.2 | 0.01 |
| Spinal cord and nerve injury | 0.2 | 0.1 | 0.00 | 0.1 | 0.1 | 0.00 |
| Traumatic brain injury (e.g., concussion) | 1.3 | 1.2 | 0.01 | 1.3 | 1.3 | 0.00 |
| **Psychiatric disorders (%)** |  |  |  |  |  |  |
| Anxiety disorder | 33.5 | 23.9 | 0.21 | 29.0 | 29.1 | 0.00 |
| Mood disorder | 0.0 | 0.0 |  | 0.0 | 0.0 |  |
| Manic episode or bipolar disorder | 0.0 | 0.0 | 0.07 | 0.0 | 0.0 | 0.00 |
| Depressive episode or major depressive disorder | 7.4 | 5.1 | 0.09 | 6.4 | 6.5 | 0.00 |
| Other mood disorder | 6.9 | 4.7 | 0.09 | 5.9 | 6.0 | 0.00 |
| **Other conditions (%)** |  |  |  |  |  |  |
| Amyloidosis | 0.1 | 0.2 | 0.02 | 0.1 | 0.1 | 0.00 |
| Biliary cirrhosis | 0.1 | 0.1 | 0.00 | 0.1 | 0.1 | 0.00 |
| Chronic kidney disease | 13.3 | 18.3 | 0.14 | 16.1 | 16.1 | 0.00 |
| Liver disease | 22.3 | 20.2 | 0.05 | 21.5 | 21.6 | 0.00 |
| Mast cell activation syndrome | 3.4 | 2.1 | 0.01 | 2.8 | 2.9 | 0.00 |
| Malignancy | 11.7 | 14.1 | 0.07 | 12.9 | 12.9 | 0.00 |
| Porphyria | 4.6 | 4.4 | 0.01 | 4.6 | 4.6 | 0.00 |
| Post-viral condition | 1.0 | 0.6 | 0.04 | 0.8 | 0.8 | 0.00 |
| Thyroid disorder | 29.3 | 25.8 | 0.08 | 27.8 | 27.7 | 0.00 |
| Weight loss surgery | 3.0 | 1.2 | 0.12 | 2.2 | 2.2 | 0.00 |
| **Baseline medication use (%)** |  |  |  |  |  |  |
| Metformin | 80.8 | 85.7 | 0.13 | 83.4 | 83.4 | 0.00 |
| Dipeptidyl peptidase-4 (DPP-4) inhibitors (gliptins) | 19.8 | 28.8 | 0.21 | 24.9 | 24.6 | 0.01 |
| Insulin therapy | 27.2 | 21.6 | 0.13 | 25.6 | 26.5 | 0.02 |
| Sulfonylureas | 30.0 | 36.3 | 0.14 | 33.9 | 34.1 | 0.00 |
| Other | 8.0 | 10.2 | 0.07 | 9.4 | 9.4 | 0.00 |
| Metoclopramide | 5.2 | 4.3 | 0.04 | 4.8 | 4.9 | 0.00 |
| Serotonin 5-HT3 antagonist antiemetics | 29.4 | 23.0 | 0.15 | 26.4 | 26.5 | 0.00 |
| Antidepressant use | 44.6 | 33.7 | 0.23 | 39.7 | 39.9 | 0.00 |
| Antipsychotics and lithium | 8.0 | 6.0 | 0.08 | 7.1 | 7.2 | 0.00 |
| Lipid lowering therapies | 71.2 | 80.1 | 0.21 | 75.6 | 76.0 | 0.01 |
| Antihypertensives | 80.5 | 84.5 | 0.11 | 82.5 | 82.7 | 0.01 |
| Prescription platelet aggregation inhibitors | 6.4 | 11.4 | 0.18 | 8.8 | 8.8 | 0.00 |
| Thiazolidinediones | 7.8 | 10.0 | 0.08 | 9.2 | 9.2 | 0.00 |
| **Healthcare utilization** |  |  |  |  |  |  |
| Hospitalizations |  |  |  |  |  |  |
| - Mean (standard deviation) | 0.1 (0.4) | 0.1 (0.5) | 0.14 | 0.1 (0.5) | 0.1 (0.4) | 0.01 |
| - Median (interquartile range) | 0 (0-0) | 0 (0-0) |  | 0 (0-0) | 0 (0-0) |  |
| - Range | 0-17 | 0-29 |  | 0-17 | 0-29 |  |
| Emergency department visits |  |  |  |  |  |  |
| - Mean (standard deviation) | 0.2 (0.6) | 0.2 (0.6) | 0.01 | 0.2 (0.7) | 0.2 (0.6) | 0.00 |
| - Median (interquartile range) | 0 (0-0) | 0 (0-0) |  | 0 (0-0) | 0 (0-0) |  |
| - Range | 0-31 | 0-41 |  | 0-31 | 0-41 |  |
| Outpatient visits |  |  |  |  |  |  |
| - Mean (standard deviation) | 13.6 (14.7) | 12.6 (13.4) | 0.07 | 13.2 (14.2) | 13.3 (14.5) | 0.01 |
| - Median (interquartile range) | 9 (5-17) | 8 (5-16) |  | 9 (5-17) | 9 (5-17) |  |
| - Range | 0-436 | 0-357 |  | 0-436 | 0-357 |  |
| Cardiologist visit on index (%) | 0.7 | 4.3 | 0.23 | 2.3 | 2.3 | 0.00 |

**Abbreviations:** ASD, absolute standardized difference; GLP-1-RA, glucagon-like peptide 1 receptor agonists; SGLT2-I, sodium glucose co-transporter 2 inhibitors; vs., versus.

# Table S3b. Covariate balance for GLP-1-RAs vs. NalBup among non-diabetic patients for chronic weight management,

# 2020-2023.

|  | **Unweighted** | | | **Weighted** | | |
| --- | --- | --- | --- | --- | --- | --- |
|  | **GLP-1-RA cohort** | **NalBup cohort** |  | **GLP-1-RA cohort** | **NalBup**  **cohort** |  |
| **Covariate** | **n=25,302** | **n=5,020** | **ASD** | **n=25,296** | **n=5,019** | **ASD** |
| **Demographics** |  |  |  |  |  |  |
| Age (Mean) | 45.4 | 44.8 | 0.05 | 45.3 | 45.2 | 0.01 |
| Sex (Female, %) | 75.7 | 83.2 | 0.19 | 77.0 | 76.7 | 0.01 |
| Region (%) |  |  | 0.34 |  |  | 0.00 |
| - Midwest | 18.6 | 26.5 | 0.19 | 19.9 | 19.8 | 0.00 |
| - Northeast | 23.1 | 11.8 | 0.30 | 21.2 | 21.4 | 0.00 |
| - South | 45.4 | 50.4 | 0.10 | 46.3 | 46.1 | 0.00 |
| - West | 12.8 | 10.9 | 0.06 | 12.5 | 12.6 | 0.00 |
| - Missing/Unknown | 0.1 | 0.4 | 0.06 | 0.1 | 0.1 | 0.00 |
| Payor commercially insured (%) | 100.0 | 100.0 | - | 100.0 | 100.0 | - |
| Index year 2020 or greater (%) | 100.0 | 100.0 | - | 100.0 | 100.0 | - |
| Race/Ethnicity (%) |  |  | 0.14 |  |  | 0.00 |
| - White, not Hispanic or Latino | 70.0 | 75.2 | 0.12 | 70.8 | 70.9 | 0.00 |
| - Black or African American, not Hispanic or Latino | 8.8 | 8.5 | 0.01 | 8.7 | 8.7 | 0.00 |
| - Hispanic or Latino, any race | 7.5 | 4.7 | 0.12 | 7.0 | 7.1 | 0.00 |
| - Asian, not Hispanic or Latino | 1.5 | 0.9 | 0.05 | 1.4 | 1.4 | 0.00 |
| - Other race, Not Hispanic or Latino | 1.3 | 1.1 | 0.02 | 1.2 | 1.1 | 0.01 |
| - Unknown or Undisclosed | 11.0 | 9.5 | 0.05 | 10.7 | 10.9 | 0.00 |
| **Prior diagnosis of a study outcome (%)** |  |  |  |  |  |  |
| Gastrointestinal conditions | 32.2 | 33.2 | 0.02 | 32.4 | 32.4 | 0.00 |
| Gastroparesis | 0.4 | 0.4 | 0.01 | 0.4 | 0.4 | 0.00 |
| Bowel obstruction | 1.5 | 1.5 | 0.00 | 1.5 | 1.5 | 0.00 |
| Gall bladder and biliary disease | 8.2 | 8.9 | 0.03 | 8.3 | 8.5 | 0.01 |
| Acute pancreatitis | 0.8 | 0.8 | 0.01 | 0.8 | 0.7 | 0.01 |
| Myocardial infarction or stroke | 1.9 | 1.6 | 0.02 | 1.8 | 1.8 | 0.01 |
| Thyroid cancer | 0.6 | 0.7 | 0.01 | 0.7 | 0.6 | 0.01 |
| Acute liver injury | 1.4 | 1.3 | 0.00 | 1.3 | 1.3 | 0.00 |
| Psychiatric hospitalization | 1.3 | 1.3 | 0.00 | 1.3 | 1.4 | 0.01 |
| Self-harm, suicidal ideation, or suicide attempt | 0.9 | 0.9 | 0.01 | 0.9 | 0.9 | 0.00 |
| **Contraindications for any study drug (%)** |  |  |  |  |  |  |
| Multiple endocrine neoplasia (MEN) type II | 0.0 | 0.0 | 0.01 | 0.0 | 0.0 | 0.00 |
| **Metabolic conditions (%)** |  |  |  |  |  |  |
| Overweight | 22.9 | 24.1 | 0.03 | 23.1 | 22.8 | 0.01 |
| Obese | 96.4 | 96.9 | 0.03 | 96.5 | 96.7 | 0.01 |
| Hypertension | 47.6 | 43.0 | 0.09 | 46.8 | 47.0 | 0.00 |
| Dyslipidemia | 52.2 | 45.1 | 0.14 | 51.0 | 50.4 | 0.01 |
| **Autoimmune conditions (%)** |  |  |  |  |  |  |
| Ankylosing spondylitis | 0.4 | 0.4 | 0.01 | 0.4 | 0.6 | 0.02 |
| Bechet’s disease | 0.0 | 0.0 | 0.02 | 0.0 | 0.1 | 0.01 |
| Celiac | 0.9 | 0.7 | 0.02 | 0.8 | 0.8 | 0.00 |
| Guillain-Barre syndrome (GBS) | 0.1 | 0.0 | 0.03 | 0.0 | 0.0 | 0.03 |
| Lupus | 1.0 | 0.9 | 0.01 | 1.0 | 1.1 | 0.01 |
| Myositis | 6.2 | 7.3 | 0.04 | 6.4 | 6.4 | 0.00 |
| Psoriasis | 4.2 | 3.7 | 0.03 | 4.1 | 4.2 | 0.00 |
| Rheumatoid arthritis | 3.5 | 3.6 | 0.01 | 3.5 | 3.5 | 0.00 |
| Sjogren’s syndrome | 0.8 | 0.9 | 0.00 | 0.9 | 0.9 | 0.00 |
| Systemic sclerosis | 0.1 | 0.1 | 0.01 | 0.1 | 0.1 | 0.01 |
| **Cardiovascular disease (%)** |  |  |  |  |  |  |
| Atherosclerotic cardiovascular disease (ASCVD) | 10.3 | 7.6 | 0.09 | 9.9 | 10.1 | 0.01 |
| Heart failure | 2.0 | 1.3 | 0.06 | 1.9 | 2.1 | 0.02 |
| **Genetic conditions (%)** |  |  |  |  |  |  |
| Ehlers-Danlos syndrome | 0.2 | 0.2 | 0.01 | 0.2 | 0.2 | 0.00 |
| Myotonic or muscular dystrophy | 0.0 | 0.0 | 0.02 | 0.0 | 0.0 | 0.02 |
| **Gastrointestinal disease (%)** |  |  |  |  |  |  |
| Gastro-esophageal reflux disease | 33.8 | 34.0 | 0.00 | 33.8 | 34.2 | 0.01 |
| Inflammatory bowel disease | 9.5 | 10.3 | 0.02 | 9.7 | 9.7 | 0.00 |
| Irritable bowel syndrome | 6.9 | 7.2 | 0.01 | 7.0 | 6.9 | 0.00 |
| **Neurological conditions (%)** |  |  |  |  |  |  |
| Amyotrophic lateral sclerosis | 0.0 | 0.0 | 0.01 | 0.0 | 0.0 | 0.00 |
| Dysautonomia | 1.1 | 1.0 | 0.01 | 1.1 | 1.0 | 0.00 |
| Migraine | 18.1 | 20.0 | 0.05 | 18.4 | 18.5 | 0.00 |
| Multiple sclerosis | 0.6 | 0.7 | 0.01 | 0.7 | 0.6 | 0.00 |
| Parkinson’s | 0.1 | 0.0 | 0.04 | 0.1 | 0.0 | 0.04 |
| Sleep disorder | 38.3 | 34.8 | 0.07 | 37.8 | 38.5 | 0.01 |
| Spinal cord and nerve injury | 0.1 | 0.2 | 0.01 | 0.1 | 0.1 | 0.00 |
| Traumatic brain injury (e.g., concussion) | 1.7 | 1.3 | 0.03 | 1.6 | 1.6 | 0.00 |
| **Psychiatric disorders (%)** |  |  |  |  |  |  |
| Anxiety disorder | 49.8 | 49.7 | 0.00 | 49.8 | 50.1 | 0.01 |
| Mood disorder |  |  |  |  |  |  |
| Manic episode or bipolar disorder | 3.4 | 2.6 | 0.05 | 3.3 | 3.3 | 0.00 |
| Depressive episode or major depressive disorder | 8.7 | 8.9 | 0.01 | 8.7 | 8.9 | 0.01 |
| Other mood disorder | 8.2 | 9.0 | 0.03 | 8.4 | 8.2 | 0.01 |
| **Other conditions (%)** |  |  |  |  |  |  |
| Amyloidosis | 0.1 | 0.1 | 0.01 | 0.1 | 0.1 | 0.00 |
| Biliary cirrhosis | 0.1 | 0.1 | 0.01 | 0.1 | 0.1 | 0.00 |
| Chronic kidney disease | 3.0 | 1.9 | 0.07 | 2.8 | 3.0 | 0.01 |
| Liver disease | 14.2 | 11.2 | 0.09 | 13.8 | 14.0 | 0.01 |
| Mast cell activation syndrome | 0.1 | 0.0 | 0.02 | 0.1 | 0.0 | 0.01 |
| Malignancy | 8.3 | 7.4 | 0.03 | 8.1 | 7.8 | 0.01 |
| Porphyria | 4.6 | 4.4 | 0.01 | 4.6 | 4.9 | 0.01 |
| Post-viral condition | 1.7 | 1.1 | 0.05 | 1.6 | 1.5 | 0.01 |
| Thyroid disorder | 28.6 | 28.0 | 0.01 | 28.5 | 28.6 | 0.00 |
| Weight loss surgery | 6.7 | 6.9 | 0.01 | 6.7 | 7.5 | 0.03 |
| **Baseline medication use (%)** |  |  |  |  |  |  |
| Metoclopramide | 5.3 | 4.4 | 0.04 | 5.2 | 5.1 | 0.00 |
| Serotonin 5-HT3 antagonist antiemetics | 37.2 | 38.5 | 0.03 | 37.4 | 37.9 | 0.01 |
| Antidepressant use | 54.5 | 56.1 | 0.03 | 54.8 | 55.3 | 0.01 |
| Antipsychotics and lithium | 8.5 | 7.5 | 0.04 | 8.4 | 8.6 | 0.01 |
| Lipid lowering therapies | 23.1 | 19.5 | 0.09 | 22.5 | 22.2 | 0.01 |
| Antihypertensives | 49.3 | 46.3 | 0.06 | 48.8 | 48.8 | 0.00 |
| Prescription platelet aggregation inhibitors | 1.3 | 0.8 | 0.05 | 1.2 | 1.1 | 0.01 |
| **Healthcare utilization** |  |  |  |  |  |  |
| Hospitalizations |  |  |  |  |  |  |
| - Mean (standard deviation) | 0.0 (0.2) | 0.0 (0.2) | 0.04 | 0.0 (0.2) | 0.0 (0.2) | 0.01 |
| - Median (interquartile range) | 0 (0-0) | 0 (0-0) |  | 0 (0-0) | 0 (0-0) |  |
| - Range | 0-4 | 0-5 |  | 0-4 | 0-5 |  |
| Emergency department visits |  |  | 0.03 |  |  |  |
| - Mean (standard deviation) | 0.1 (0.5) | 0.1 (0.4) |  | 0.1 (0.5) | 0.1 (0.4) | 0.00 |
| - Median (interquartile range) | 0 (0-0) | 0 (0-0) |  | 0 (0-0) | 0 (0-0) |  |
| - Range | 0-21 | 0-6 |  | 0-21 | 0-6 |  |
| Outpatient visits |  |  |  |  |  |  |
| - Mean (standard deviation) | 13.1 (13.0) | 12.1 (11.1) | 0.08 | 13.0 (12.8) | 12.9 (12.2) | 0.01 |
| - Median (interquartile range) | 9 (5-17) | 9 (5-16) |  | 9 (5-17) | 9 (5-16) |  |
| - Range | 0-497 | 0-124 |  | 0-497 | 0-124 |  |
| Cardiologist visit on index (%) | 1.3 | 0.4 | 0.11 | 1.2 | 1.0 | 0.01 |

**Abbreviations:** ASD, absolute standardized difference; GLP-1-RA, glucagon-like peptide 1 receptor agonists; NalBup, naltrexone hydrocholoride/bupropion hydrochloride; vs., versus.

# Table S3c. Covariate balance for GLP vs. PhenTop among non-diabetic patients for chronic weight management, 2020-2023.

|  | **Unweighted** | | | **Weighted** | | |
| --- | --- | --- | --- | --- | --- | --- |
|  | **GLP-1-RA cohort** | **PhenTop cohort** |  | **GLP-1-RA cohort** | **PhenTop cohort** |  |
| **Covariate** | **n=25,496** | **n=3,842** | **ASD** | **n=25,489** | **n=3,841** | **ASD** |
| **Demographics** |  |  |  |  |  |  |
| Age (Mean) | 45.5 | 44.4 | 0.10 | 45.3 | 45.4 | 0.00 |
| Sex (Female, %) | 75.8 | 83.1 | 0.18 | 76.7 | 76.8 | 0.00 |
| Region (%) |  |  | 0.30 |  |  | 0.00 |
| - Midwest | 18.6 | 29.9 | 0.27 | 20.1 | 20.3 | 0.01 |
| - Northeast | 23.0 | 15.7 | 0.19 | 22.1 | 21.7 | 0.01 |
| - South | 45.7 | 40.0 | 0.11 | 44.9 | 45.4 | 0.01 |
| - West | 12.7 | 14.2 | 0.04 | 12.9 | 12.5 | 0.01 |
| - Missing/Unknown | 0.1 | 0.3 | 0.05 | 0.1 | 0.1 | 0.00 |
| Payor Commercially Insured (%) | 100.0 | 100.0 | - | 100.0 | 100.0 | - |
| Index year 2020 or greater (%) | 100.0 | 100.0 | - | 100.0 | 100.0 | - |
| Race/Ethnicity (%) |  |  | 0.09 |  |  | 0.00 |
| - White, not Hispanic or Latino | 70.0 | 69.7 | 0.01 | 70.0 | 70.2 | 0.00 |
| - Black or African American, not Hispanic or Latino | 8.8 | 10.5 | 0.06 | 9.0 | 8.8 | 0.01 |
| - Hispanic or Latino, any race | 7.5 | 6.6 | 0.04 | 7.4 | 7.3 | 0.00 |
| - Asian, not Hispanic or Latino | 1.5 | 1.4 | 0.01 | 1.5 | 1.4 | 0.01 |
| - Other race, Not Hispanic or Latino | 1.2 | 1.1 | 0.01 | 1.2 | 1.2 | 0.01 |
| - Unknown or Undisclosed | 11.0 | 10.7 | 0.01 | 10.9 | 11.1 | 0.01 |
| **Prior diagnosis of a study outcome (%)** |  |  |  |  |  |  |
| Gastrointestinal conditions | 32.4 | 32.5 | 0.00 | 32.4 | 31.9 | 0.01 |
| Gastroparesis | 0.4 | 0.5 | 0.01 | 0.4 | 0.4 | 0.00 |
| Bowel obstructions | 1.5 | 1.7 | 0.02 | 1.5 | 1.5 | 0.00 |
| Gall bladder and biliary disease | 8.3 | 9.0 | 0.03 | 8.4 | 9.0 | 0.02 |
| Acute pancreatitis | 0.8 | 0.6 | 0.02 | 0.8 | 0.7 | 0.01 |
| Myocardial infarction or stroke | 1.9 | 1.0 | 0.07 | 1.8 | 2.2 | 0.03 |
| Thyroid cancer | 0.6 | 1.0 | 0.04 | 0.7 | 0.7 | 0.00 |
| Acute liver injury | 1.4 | 1.5 | 0.01 | 1.4 | 1.3 | 0.01 |
| Psychiatric hospitalization | 1.3 | 1.1 | 0.02 | 1.3 | 1.4 | 0.01 |
| Self-harm, suicidal ideation, or suicide attempt | 0.9 | 0.8 | 0.01 | 0.9 | 1.0 | 0.01 |
| **Contraindications for any study drug (%)** |  |  |  |  |  |  |
| Multiple endocrine neoplasia (MEN) type II | 0.0 | 0.0 | 0.01 | 0.0 | 0.0 | 0.01 |
| **Metabolic conditions (%)** |  |  |  |  |  |  |
| Overweight | 23.0 | 24.5 | 0.03 | 23.2 | 23.3 | 0.00 |
| Obese | 96.4 | 96.4 | 0.00 | 96.4 | 96.4 | 0.00 |
| Hypertension | 47.7 | 36.5 | 0.23 | 46.3 | 46.6 | 0.01 |
| Dyslipidemia | 52.3 | 43.6 | 0.17 | 51.1 | 51.4 | 0.01 |
| **Autoimmune conditions (%)** |  |  |  |  |  |  |
| Ankylosing spondylitis | 0.4 | 0.4 | 0.00 | 0.4 | 0.6 | 0.03 |
| Bechet’s disease | 0.0 | 0.1 | 0.01 | 0.1 | 0.0 | 0.00 |
| Celiac | 0.9 | 0.8 | 0.01 | 0.8 | 0.8 | 0.01 |
| Guillain-Barre syndrome (GBS) | 0.1 | 0.0 | 0.03 | 0.1 | 0.0 | 0.03 |
| Lupus | 1.0 | 1.0 | 0.00 | 1.0 | 1.0 | 0.00 |
| Myositis | 6.3 | 6.1 | 0.01 | 6.3 | 6.2 | 0.00 |
| Psoriasis | 4.2 | 3.3 | 0.05 | 4.1 | 3.9 | 0.01 |
| Rheumatoid arthritis | 3.5 | 2.7 | 0.05 | 3.4 | 3.4 | 0.00 |
| Sjogren’s syndrome | 0.9 | 1.0 | 0.01 | 0.9 | 0.9 | 0.00 |
| Systemic sclerosis | 0.1 | 0.0 | 0.04 | 0.1 | 0.1 | 0.02 |
| **Cardiovascular disease (%)** |  |  |  |  |  |  |
| Atherosclerotic cardiovascular disease (ASCVD) | 10.3 | 5.6 | 0.18 | 9.7 | 10.6 | 0.03 |
| Heart failure | 2.0 | 0.6 | 0.13 | 1.8 | 2.0 | 0.01 |
| **Genetic disorders (%)** |  |  |  |  |  |  |
| Ehlers-Danlos syndrome | 0.2 | 0.2 | 0.01 | 0.2 | 0.2 | 0.01 |
| Myotonic or muscular dystrophy | 0.0 | 0.1 | 0.01 | 0.0 | 0.0 | 0.01 |
| **Gastrointestinal disease (%)** |  |  |  |  |  |  |
| Gastro-esophageal reflux disease | 34.0 | 30.9 | 0.07 | 33.6 | 33.9 | 0.01 |
| Inflammatory bowel disease | 9.6 | 9.1 | 0.02 | 9.5 | 10.0 | 0.02 |
| Irritable bowel syndrome | 7.0 | 6.9 | 0.00 | 6.9 | 6.7 | 0.01 |
| **Neurological conditions (%)** |  |  |  |  |  |  |
| Amyotrophic lateral sclerosis | 0.0 | 0.0 | 0.01 | 0.0 | 0.0 | 0.01 |
| Dysautonomia | 1.1 | 0.7 | 0.04 | 1.1 | 1.0 | 0.01 |
| Migraine | 18.2 | 19.2 | 0.02 | 18.3 | 18.2 | 0.00 |
| Multiple sclerosis | 0.6 | 0.5 | 0.01 | 0.6 | 0.6 | 0.00 |
| Parkinson’s | 0.1 | 0.0 | 0.03 | 0.1 | 0.3 | 0.05 |
| Sleep disorders | 38.4 | 32.5 | 0.13 | 37.7 | 38.6 | 0.02 |
| Spinal cord and nerve injury | 0.1 | 0.2 | 0.01 | 0.1 | 0.1 | 0.01 |
| Traumatic brain injury (e.g., concussion) | 1.7 | 1.4 | 0.03 | 1.6 | 1.6 | 0.00 |
| **Psychiatric disorders (%)** |  |  |  |  |  |  |
| Anxiety disorder | 49.9 | 48.3 | 0.03 | 49.7 | 49.0 | 0.01 |
| Mood disorder |  |  |  |  |  |  |
| Manic episode or bipolar disorder | 3.4 | 2.9 | 0.03 | 3.3 | 3.5 | 0.01 |
| Depressive episode or major depressive disorder | 8.8 | 8.6 | 0.01 | 8.8 | 8.8 | 0.00 |
| Other mood disorder | 8.3 | 8.4 | 0.00 | 8.3 | 8.2 | 0.00 |
| **Other conditions (%)** |  |  |  |  |  |  |
| Amyloidosis | 0.1 | 0.0 | 0.02 | 0.1 | 0.2 | 0.04 |
| Biliary cirrhosis | 0.1 | 0.1 | 0.01 | 0.1 | 0.1 | 0.02 |
| Chronic kidney disease | 3.0 | 1.6 | 0.10 | 2.8 | 3.1 | 0.02 |
| Liver disease | 14.2 | 11.1 | 0.09 | 13.8 | 13.6 | 0.01 |
| Mast cell activation syndrome | 0.1 | 0.1 | 0.01 | 0.1 | 0.1 | 0.01 |
| Malignancy | 8.3 | 7.1 | 0.05 | 8.2 | 7.6 | 0.02 |
| Porphyria | 4.7 | 4.6 | 0.00 | 4.7 | 5.0 | 0.02 |
| Post-viral condition | 1.7 | 0.9 | 0.07 | 1.6 | 1.9 | 0.02 |
| Thyroid disorder | 28.7 | 28.9 | 0.00 | 28.7 | 29.0 | 0.01 |
| Weight loss surgery | 6.7 | 7.0 | 0.01 | 6.8 | 7.1 | 0.01 |
| **Baseline medication use (%)** |  |  |  |  |  |  |
| Metoclopramide | 5.4 | 5.4 | 0.00 | 5.4 | 5.6 | 0.01 |
| Serotonin 5-HT3 antagonist antiemetics | 37.4 | 35.7 | 0.04 | 37.2 | 37.5 | 0.01 |
| Antidepressant use | 54.7 | 52.7 | 0.04 | 54.5 | 55.0 | 0.01 |
| Antipsychotics and lithium | 8.6 | 6.9 | 0.06 | 8.4 | 8.9 | 0.02 |
| Lipid lowering therapies | 23.2 | 17.1 | 0.15 | 22.4 | 22.9 | 0.01 |
| Antihypertensives | 49.5 | 40.1 | 0.19 | 48.3 | 48.2 | 0.00 |
| Prescription platelet aggregation inhibitors | 1.3 | 0.4 | 0.09 | 1.2 | 1.6 | 0.03 |
| **Healthcare utilization** |  |  |  |  |  |  |
| Hospitalizations |  |  |  |  |  |  |
| - Mean (standard deviation) | 0.0 (0.2) | 0.0 (0.2) | 0.05 | 0.03 (0.2) | 0.03 (0.2) | 0.01 |
| - Median (interquartile range) | 0 (0-0) | 0 (0-0) |  | 0 (0-0) | 0 (0-0) |  |
| - Range | 0-4 | 0-3 |  | 0-4 | 0-3 |  |
| Emergency department visits |  |  |  |  |  |  |
| - Mean (standard deviation) | 0.1 (0.5) | 0.1 (0.4) | 0.06 | 0.13 (0.4) | 0.15 (0.6) | 0.04 |
| - Median (interquartile range) | 0 (0-0) | 0 (0-0) |  | 0 (0-0) | 0 (0-0) |  |
| - Range | 0-21 | 0-8 |  | 0-21 | 0-8 |  |
| Outpatient visits |  |  |  |  |  |  |
| - Mean (standard deviation) | 13.2 (13.0) | 12.3 (11.4) | 0.07 | 13.0 (13.0) | 13.1 (12.2) | 0.000 |
| - Median (interquartile range) | 9 (5-17) | 9 (5-16) |  | 9 (5-17) | 9 (5-17) |  |
| - Range | 0-497 | 0-128 |  | 0-497 | 0-128 |  |
| Cardiologist visit on index (%) | 1.3 | 0.4 | 0.11 |  |  | 0.01 |

**Abbreviations:** ASD, absolute standardized difference; GLP-1-RA, glucagon-like peptide 1 receptor agonists; PhenTop, phentermine/topiramate extended-release vs., versus.

# Table S4a. Incidence, rate ratio, and NNT/H estimates for study outcomes for GLP-1-RAs vs. SGLT2-I among patients with type 2 diabetes, 2016-2023.

|  |  | **GLP-1-RA cohort** | | | **SGLT2-I cohort** | | |  |  |  |
| --- | --- | --- | --- | --- | --- | --- | --- | --- | --- | --- |
| **Outcome** | **Analysis^1^** | **n** | **Person-Years^2^** | **Rate^3^** | **n** | **Person-Years^2^** | **Rate^3^** | **Rate Ratio**  **(95% CI)** | **6-month**  **RD^4^** | **6-month NNT/H^4^** |
| GI hospitalization | Unweighted | 2,584.0 | 299,438.3 | 8.63 | 2,285.0 | 281,341.3 | 8.12 | 1.06 (1.00, 1.12) | -0.0004 | 2,316 |
|  | Weighted incl. A1c | 886.3 | 101,645.6 | 8.72 | 612.1 | 87,253.3 | 7.01 | 1.24 (1.12, 1.38) | 0.0005 | 2,201 |
| Gastroparesis hospitalization | Unweighted | 82.0 | 301,107.1 | 0.27 | 60.0 | 282,885.9 | 0.21 | 1.28 (0.92, 1.79) | <0.0001 | 73,278 |
|  | Weighted incl. A1c | 22.8 | 102,252.8 | 0.22 | 19.8 | 87,680.9 | 0.23 | 0.99 (0.54, 1.8) | <0.0001 | 32,529 |
| Bowel obstruction hospitalization | Unweighted | 687.0 | 300,655.1 | 2.29 | 658.0 | 282,487.7 | 2.33 | 0.98 (0.88, 1.09) | -0.0002 | 5,218 |
|  | Weighted incl. A1c | 215.4 | 102,098.8 | 2.11 | 179.6 | 87,550.1 | 2.05 | 1.03 (0.84, 1.25) | <0.0001 | 27,299 |
| Gall bladder and biliary disease hospitalization | Unweighted | 1,470.0 | 300,139.3 | 4.90 | 1,471.0 | 281,877.9 | 5.22 | 0.94 (0.87, 1.01) | -0.0006 | 1,722 |
|  | Weighted incl. A1c | 491.4 | 101,920.6 | 4.82 | 390.7 | 87,412.0 | 4.47 | 1.08 (0.94, 1.23) | -0.0002 | 4,023 |
| Acute pancreatitis hospitalization | Unweighted | 558.0 | 300,826.5 | 1.85 | 615.0 | 282,369.4 | 2.18 | 0.85 (0.76, 0.96) | -0.0005 | 2,206 |
|  | Weighted incl. A1c | 186.7 | 102,157.0 | 1.83 | 138.9 | 87,564.6 | 1.59 | 1.15 (0.93, 1.44) | <0.0001 | 21,168 |
| MI or stroke hospitalization | Unweighted | 3,321.0 | 298,654.1 | 11.12 | 4,132.0 | 279,697.6 | 14.77 | 0.75 (0.72, 0.79) | -0.0032 | 313 |
|  | Weighted incl. A1c | 1,277.0 | 101,240.5 | 12.61 | 1,014.2 | 86,854.7 | 11.68 | 1.08 (0.99, 1.17) | -0.0001 | 6,909 |
| Thyroid cancer^5^ | Unweighted | 828.0 | 301,152.6 | 2.75 | 708.0 | 282,947.2 | 2.50 | 1.1 (0.99, 1.21) | -0.0001 | 11,329 |
|  | Weighted incl. A1c | 275.0 | 102,265.3 | 2.69 | 244.9 | 87,689.1 | 2.79 | 0.96 (0.81, 1.14) | -0.0002 | 4,244 |
| Acute liver injury hospitalization | Unweighted | 197.0 | 301,058.5 | 0.65 | 283.0 | 282,823.3 | 1.00 | 0.65 (0.55, 0.78) | -0.0002 | 4,108 |
|  | Weighted incl. A1c | 54.6 | 102,248.7 | 0.53 | 77.7 | 87,656.4 | 0.89 | 0.6 (0.43, 0.85) | -0.0003 | 2,998 |
| Psychiatric hospitalization | Unweighted | 1,319.0 | 300,261.8 | 4.39 | 1,106.0 | 282,181.0 | 3.92 | 1.12 (1.03, 1.21) | <0.0001 | 48,151 |
|  | Weighted incl. A1c | 400.8 | 102,008.2 | 3.93 | 360.0 | 87,421.5 | 4.12 | 0.95 (0.83, 1.1) | 0.0001 | 19,711 |
| Suicidal ideation or self-harm | Unweighted | 810.0 | 300,586.5 | 2.69 | 580.0 | 282,506.1 | 2.05 | 1.31 (1.18, 1.46) | 0.0002 | 4,154 |
|  | Weighted incl. A1c | 237.5 | 102,108.9 | 2.33 | 199.4 | 87,521.4 | 2.28 | 1.02 (0.85, 1.23) | 0.0001 | 12,489 |
| All-cause mortality | Unweighted | 1,682.0 | 301,152.6 | 5.59 | 2,573.0 | 282,947.2 | 9.09 | 0.61 (0.58, 0.65) | -0.0031 | 319 |
|  | Weighted incl. A1c | 633.9 | 102,265.3 | 6.20 | 517.4 | 87,689.1 | 5.90 | 1.05 (0.94, 1.18) | -0.0004 | 2,546 |

**Abbreviations**: CI, confidence interval; GI, gastrointestinal; MI, myocardial infarction; n, number of patients who experienced the outcome during follow-up; NNT/H, number needed to treat to see benefit or harm.

^1^The weighted analysis adjusted for 82 clinical and demographic covariates.

^2^Patients were followed until pregnancy, death, disenrollment, malignancy, bariatric surgery, discontinuation of their index medication, initiation of an SGLT2-I (GLP-1-RA users only) or a GLP-1-RA (SGLT2-I users only), occurrence of the outcome of interest, or end of available data (12/31/2023).

^3^Rate per 1,000 person-years.

^4^NNT/Hs were calculated using the Kaplan-Meier-based approach described by Suissa (2015; PMID 26241223). The 6-month time point was selected since mean and median follow-up time were between 6 and 12 months.

^5^Two outpatient visits at least 30 days apart or one inpatient visit with a primary diagnosis code for thyroid cancer.

# Table S4b. Incidence, rate ratio, and NNT/H estimates for study outcomes for GLP-1-RAs vs. NalBup among patients without prediabetes or diabetes for chronic weight management, 2020-2023.

|  |  | **GLP-1-RA cohort** | | | **NalBup cohort** | | |  |  |  |
| --- | --- | --- | --- | --- | --- | --- | --- | --- | --- | --- |
| **Outcome** | **Analysis^1^** | **n** | **Person-Years^2^** | **Rate^3^** | **n** | **Person-Years^2^** | **Rate^3^** | **Rate Ratio (95% CI)** | **6-month**  **RD^4^** | **6-month NNT/H^4^** |
| GI hospitalization | Unweighted | 106.0 | 17,533.1 | 6.05 | 25.0 | 4,232.4 | 5.91 | 0.99 (0.64, 1.53) | 0.0002 | 5,343 |
| Gastroparesis hospitalization | Unweighted | 2.0 | 17,577.6 | 0.11 | - | 4,247.2 | - | - | 0.0001 | 10,945 |
| Bowel obstruction hospitalization | Unweighted | 29.0 | 17,565.1 | 1.65 | 5.0 | 4,241.2 | 1.18 | 1.4 (0.54, 3.62) | <0.0001 | 24,382 |
| Gall bladder and biliary disease hospitalization | Unweighted | 84.0 | 17,543.9 | 4.79 | 6.0 | 4,241.1 | 1.41 | 3.38 (1.48, 7.75) | 0.0011 | 875 |
| Acute pancreatitis hospitalization | Unweighted | 22.0 | 17,566.6 | 1.25 | 1.0 | 4,247.1 | 0.24 | 5.32 (0.72, 39.46) | 0.0005 | 2,221 |
| MI or stroke hospitalization | Unweighted | 36.0 | 17,559.9 | 2.05 | 7.0 | 4,236.5 | 1.65 | 1.24 (0.55, 2.79) | 0.0003 | 3,319 |
| Thyroid cancer^5^ | Unweighted | 87.0 | 17,578.0 | 4.95 | 15.0 | 4,247.2 | 3.53 | 1.4 (0.81, 2.42) | 0.0002 | 4,336 |
| Acute liver injury hospitalization | Unweighted | 6.0 | 17,576.6 | 0.34 | - | 4,247.2 | - | - | 0.0001 | 11,513 |
| Psychiatric hospitalization | Unweighted | 48.0 | 17,555.2 | 2.73 | 12.0 | 4,238.0 | 2.83 | 0.97 (0.51, 1.82) | -0.0001 | 12,500 |
| Suicidal ideation or self harm | Unweighted | 37.0 | 17,559.9 | 2.11 | 15.0 | 4,234.3 | 3.54 | 0.59 (0.33, 1.08) | -0.0012 | 864 |
| All-cause mortality | Unweighted | 11.0 | 17,578.0 | 0.63 | 2.0 | 4,247.2 | 0.47 | 1.33 (0.29, 6) | -0.0003 | 3,652 |

**Abbreviations**: CI, confidence interval; GI, gastrointestinal; MI, myocardial infarction; n, number of patients who experienced the outcome during follow-up; NNT/H, number needed to treat to see benefit or harm.

^1^The weighted analysis adjusted for 82 clinical and demographic covariates.

^2^Patients were followed until pregnancy, death, disenrollment, malignancy, bariatric surgery, discontinuation of their index medication, initiation of NalBup (GLP-1-RA users only) or a GLP-1-RA (NalBup users only), occurrence of the outcome of interest, or end of available data (12/31/2023).

^3^Rate per 1,000 person-years.

^4^NNT/Hs were calculated using the Kaplan-Meier-based approach described by Suissa (2015; PMID 26241223). The 6-month time point was selected since mean and median follow-up time were between 6 and 12 months.

^5^Two outpatient visits at least 30 days apart or one inpatient visit with a primary diagnosis code for thyroid cancer.

# Table S4c. Incidence, rate ratio, and NNT/H estimates for study outcomes for GLP-1-RAs vs. PhenTop among patients without prediabetes or diabetes for chronic weight management, 2020-2023.

|  |  | **GLP-1-RA cohort** | | | **PhenTop cohort** | | |  |  |  |
| --- | --- | --- | --- | --- | --- | --- | --- | --- | --- | --- |
| **Outcome** | **Analysis^1^** | **n** | **Person-Years^2^** | **Rate^3^** | **n** | **Person-Years^2^** | **Rate^3^** | **Rate Ratio (95% CI)** | **6-month**  **RD^4^** | **6-month NNT/H^4^** |
| GI hospitalization | Unweighted | 108 | 17,691.8 | 6.1 | 15 | 2,392.5 | 6.3 | 1.0 (0.6, 1.7) | -0.0004 | 2,344 |
| Gastroparesis hospitalization | Unweighted | 2 | 17,736.8 | 0.1 | 1 | 2,395.4 | 0.4 | 0.3 (0.0, 3.0) | -0.0004 | 2,398 |
| Bowel obstruction hospitalization | Unweighted | 29 | 17,724.2 | 1.6 | 2 | 2,394.2 | 0.8 | 2.0 (0.5, 8.2) | 0.0003 | 3,368 |
| Gall bladder and biliary disease hospitalization | Unweighted | 85 | 17,701.9 | 4.8 | 3 | 2,394.6 | 1.3 | 3.8 (1.2, 12.1) | 0.0010 | 990 |
| Acute pancreatitis hospitalization | Unweighted | 22 | 17,725.7 | 1.2 | 2 | 2,393.1 | 0.8 | 1.5 (0.3, 6.3) | 0.0007 | 1,502 |
| MI or stroke Hospitalization | Unweighted | 38 | 17,717.4 | 2.1 | 2 | 2,395.0 | 0.8 | 2.6 (0.6, 10.6) | 0.0010 | 968 |
| Thyroid cancer^5^ | Unweighted | 86 | 17,737.1 | 4.8 | 13 | 2,395.6 | 5.4 | 0.9 (0.5, 1.6) | -0.0014 | 710 |
| Acute liver injury Hospitalization | Unweighted | 6 | 17,735.8 | 0.3 | 0 | 2,395.6 | 0.0 | - | 0.0001 | 11,605 |
| Psychiatric hospitalization | Unweighted | 48 | 17,714.3 | 2.7 | 4 | 2,394.2 | 1.7 | 1.6 (0.6, 4.5) | 0.0003 | 3,605 |
| Suicidal ideation or self harm | Unweighted | 37 | 17,719.0 | 2.1 | 4 | 2,395.0 | 1.7 | 1.3 (0.4, 3.5) | 0.0007 | 1,382 |
| All-cause mortality | Unweighted | 11 | 17,737.1 | 0.6 | 0 | 2,395.6 | 0.0 | - | 0.0003 | 3,997 |

**Abbreviations**: CI, confidence interval; GI, gastrointestinal; MI, myocardial infarction; n, number of patients who experienced the outcome during follow-up; NNT/H, number needed to treat to see benefit or harm.

^1^The weighted analysis adjusted for 82 clinical and demographic covariates.

^2^Patients were followed until pregnancy, death, disenrollment, malignancy, bariatric surgery, discontinuation of their index medication, initiation of PhenTop (GLP-1-RA users only) or a GLP-1-RA (PhenTop users only), occurrence of the outcome of interest, or end of available data (12/31/2023).

^3^Rate per 1,000 person-years.

^4^NNT/Hs were calculated using the Kaplan-Meier-based approach described by Suissa (2015; PMID 26241223). The 6-month time point was selected since mean and median follow-up time were between 6 and 12 months.

^5^Two outpatient visits at least 30 days apart or one inpatient visit with a primary diagnosis code for thyroid cancer.

# Figure S1. Weighted Kaplan-Meier Plots – Type 2 Diabetes Analysis.


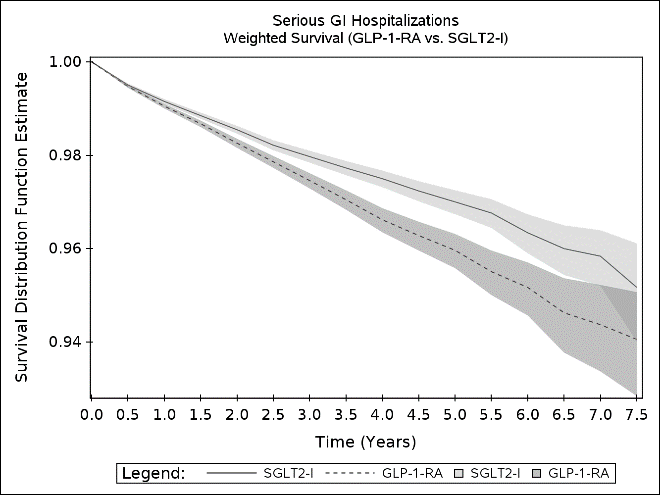

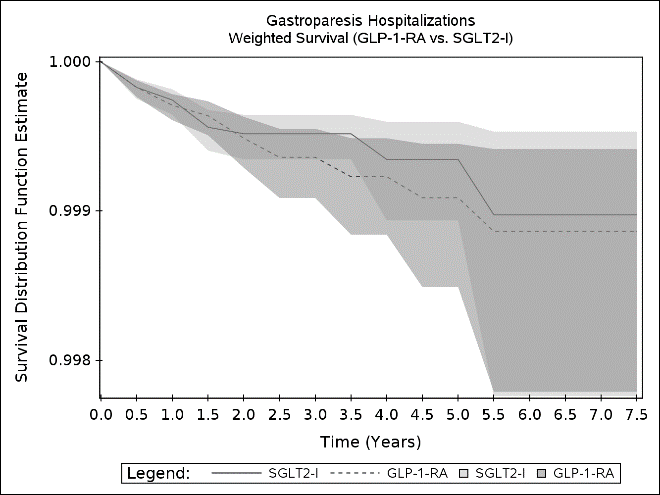

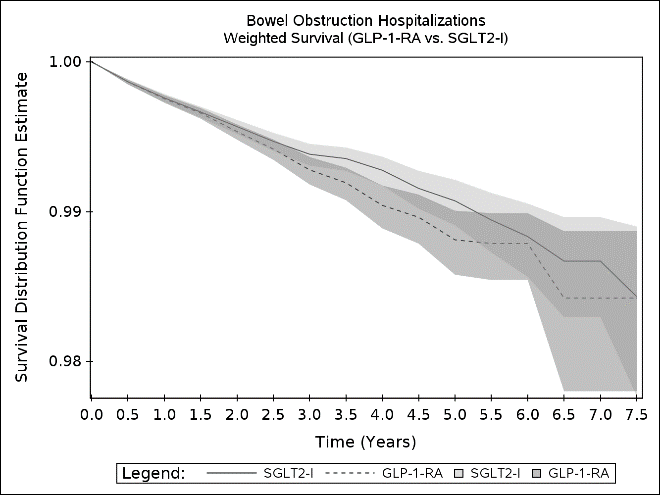

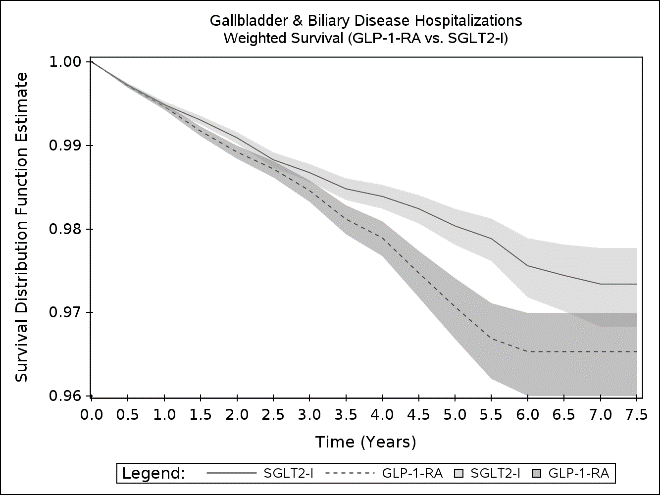

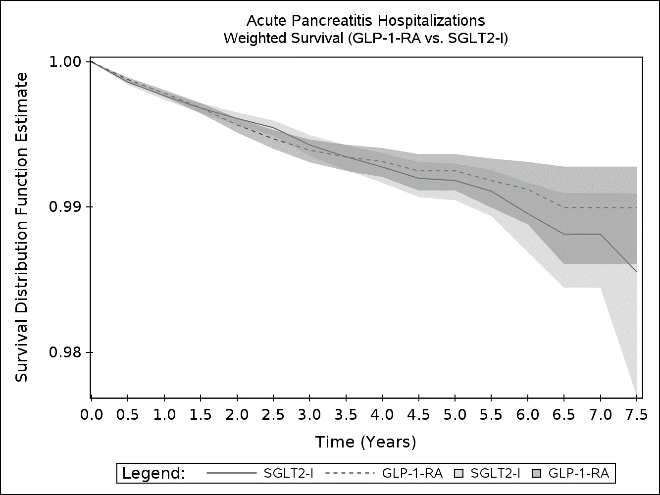

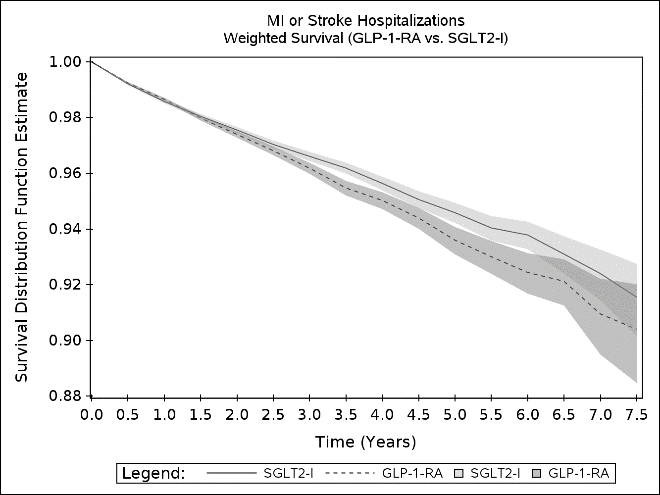

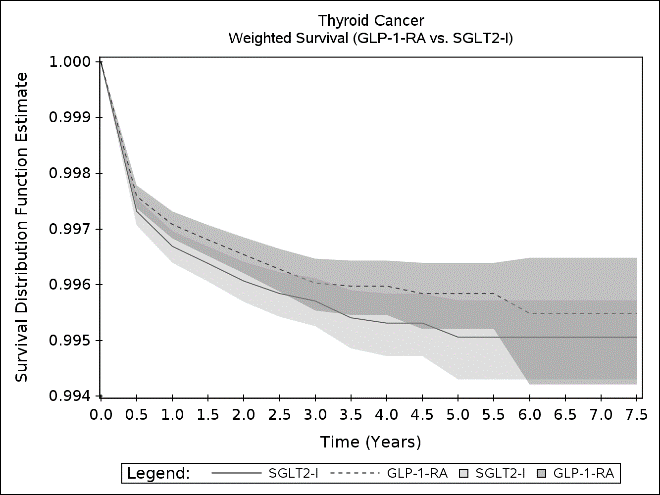

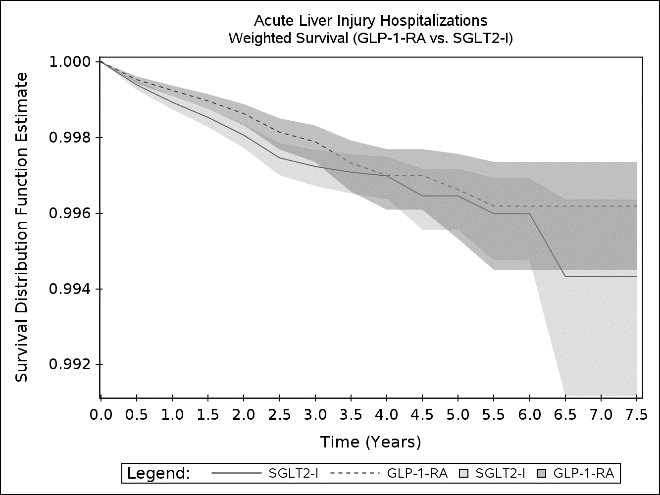

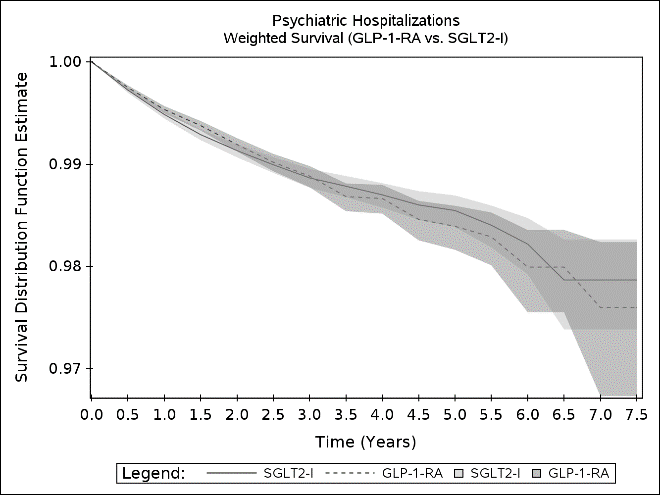

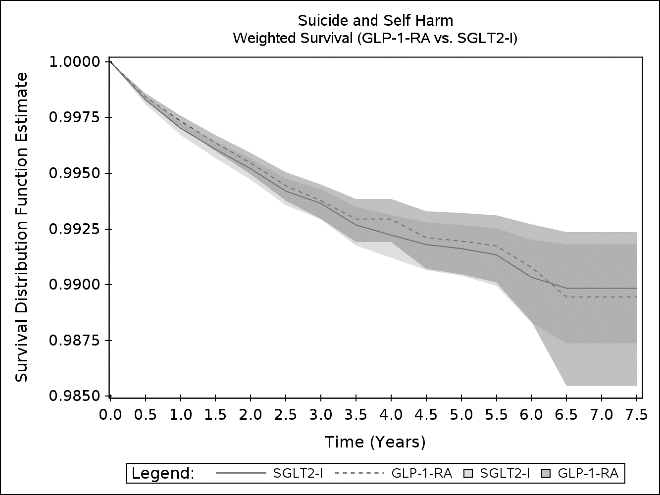

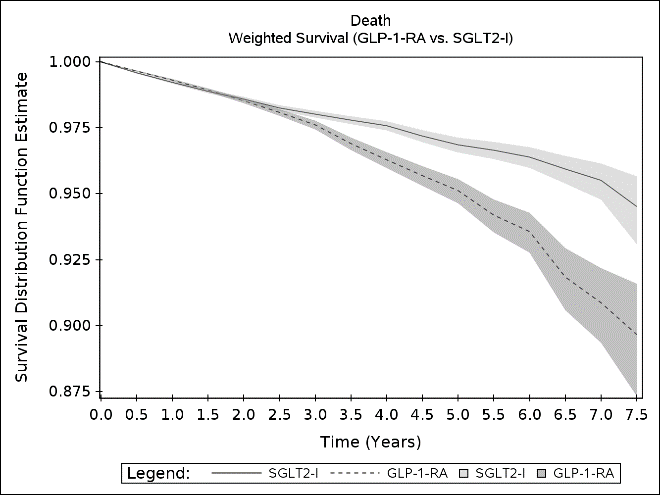


# Figure S2. Weighted Kaplan-Meier Plots – Chronic Weight Management Analyses with NalBup as comparator.


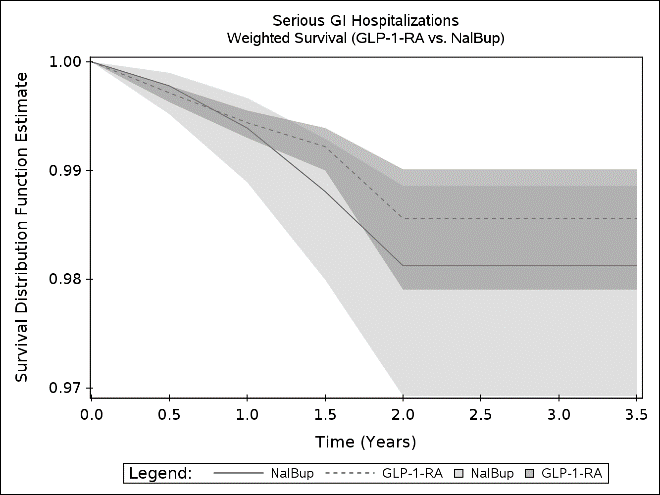

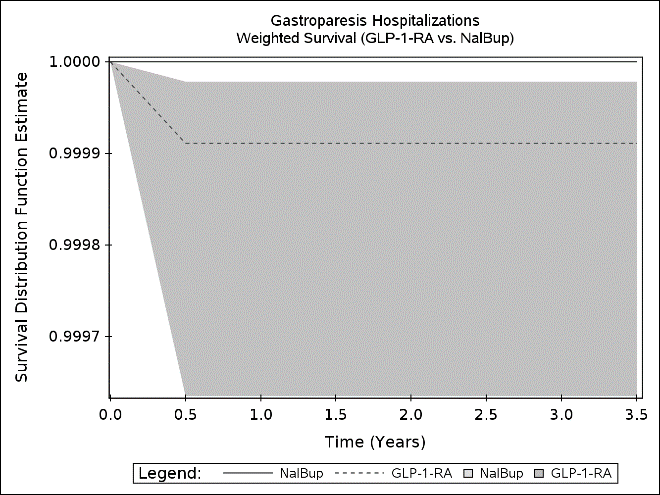

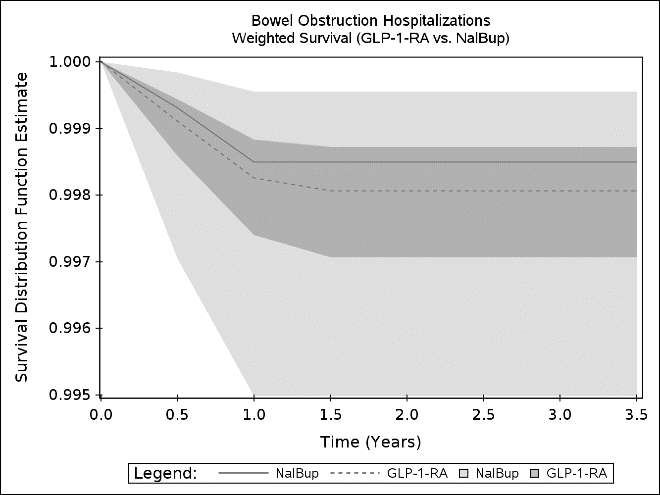

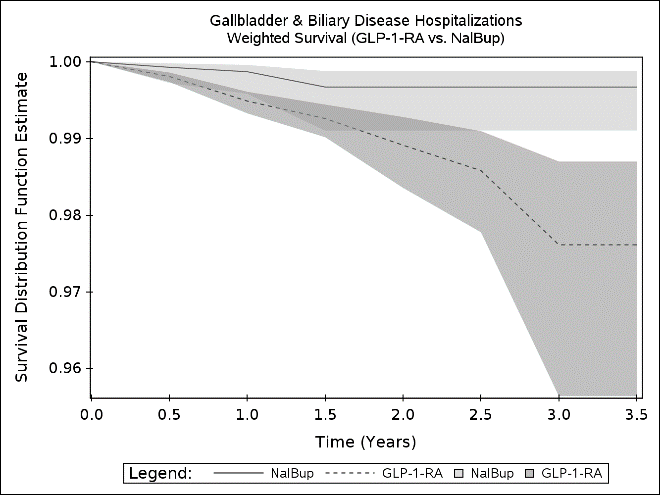

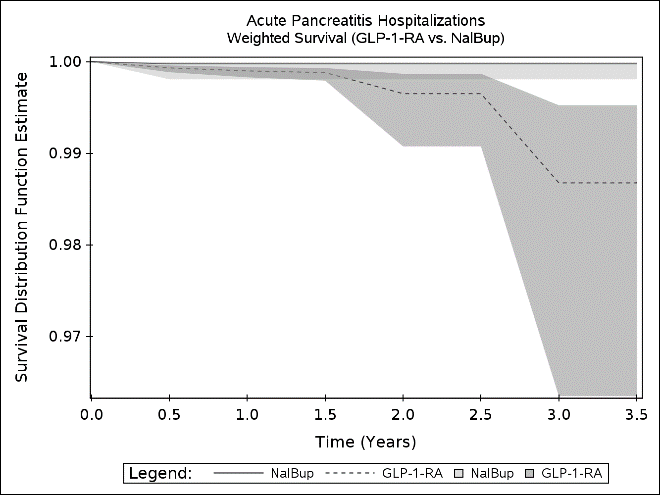

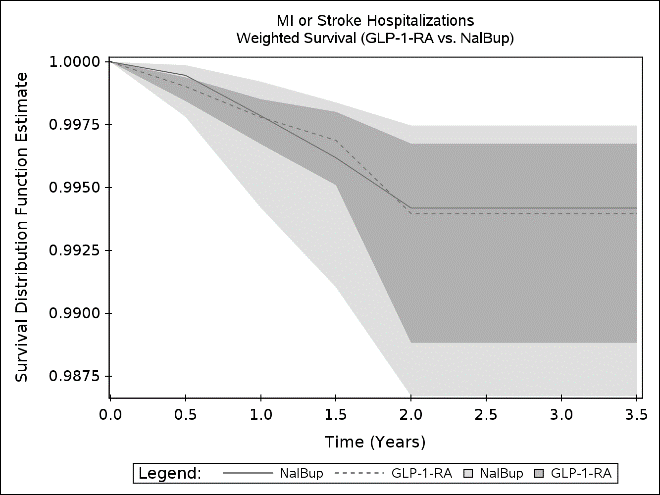

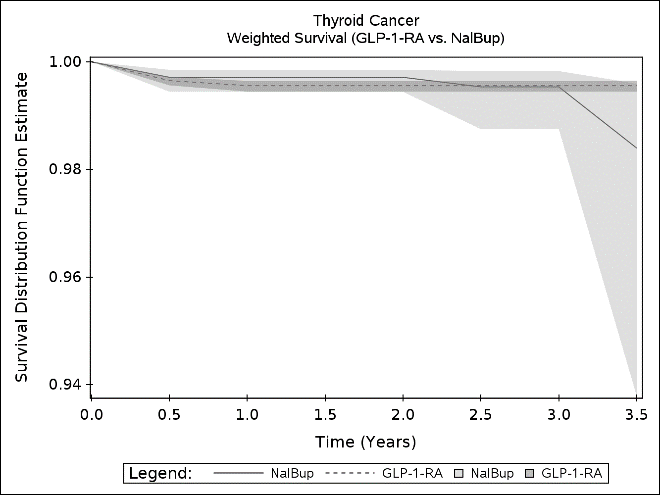

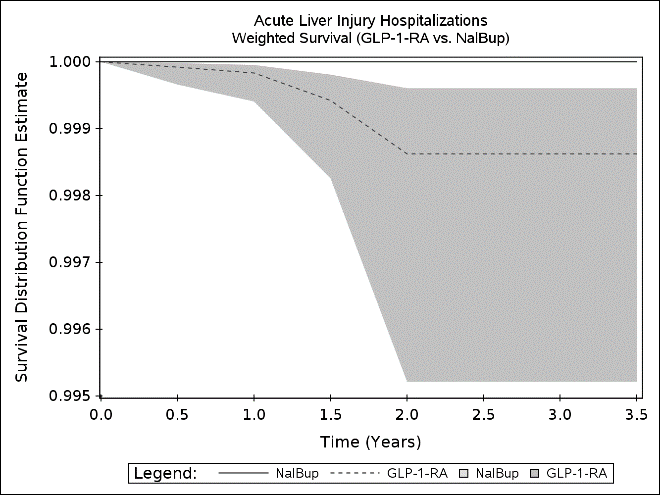

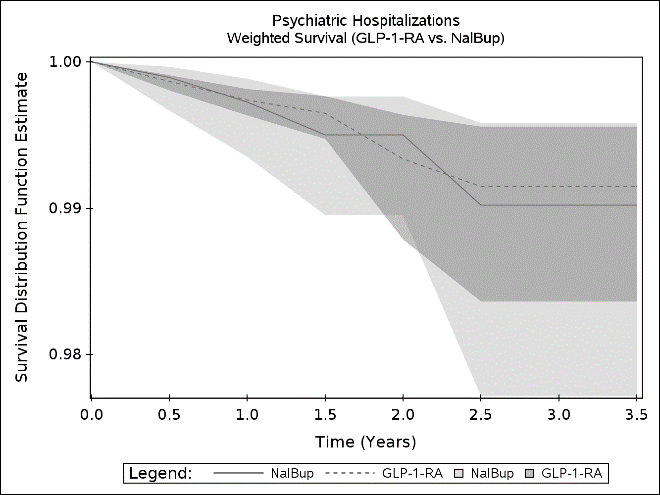

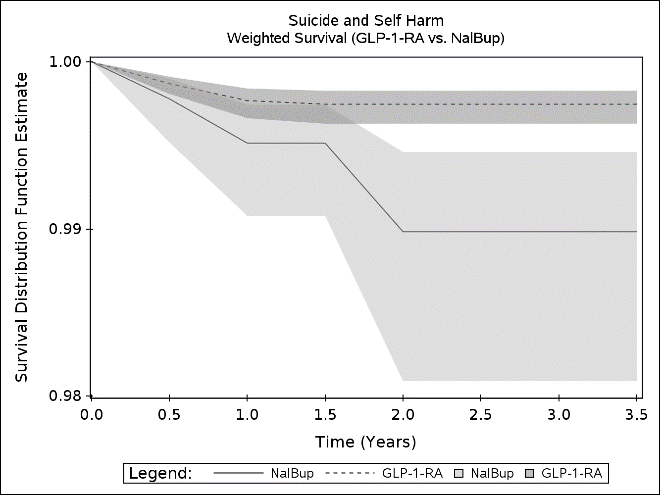

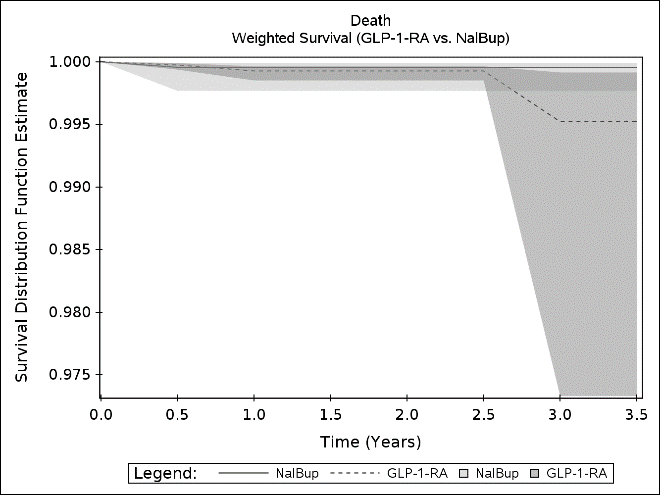


# Figure S3. Weighted Kaplan-Meier Plots – Chronic Weight Management Analyses with PhenTop as comparator.


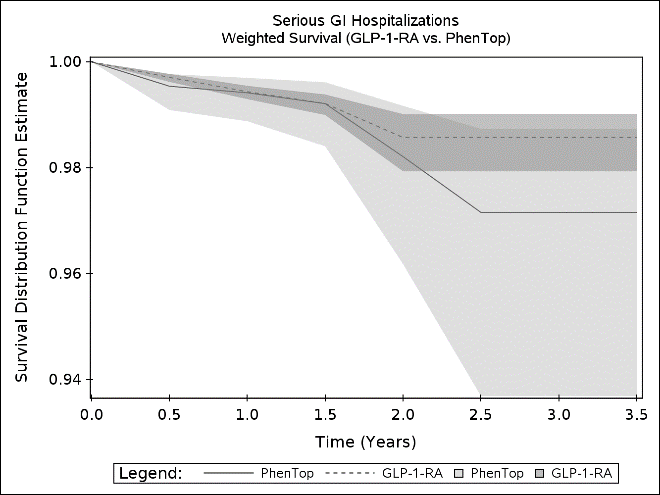

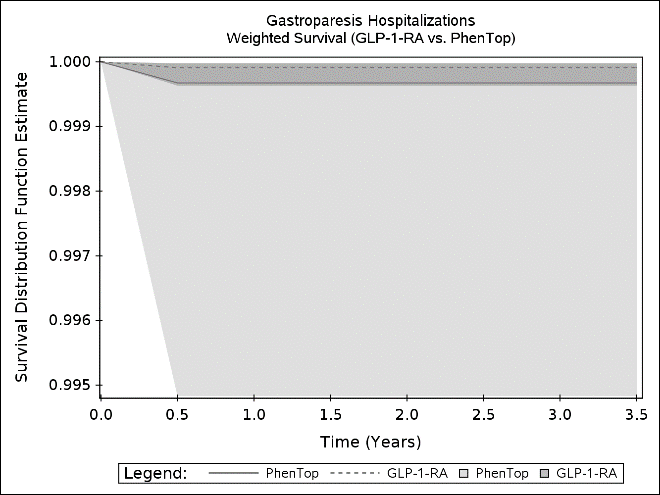

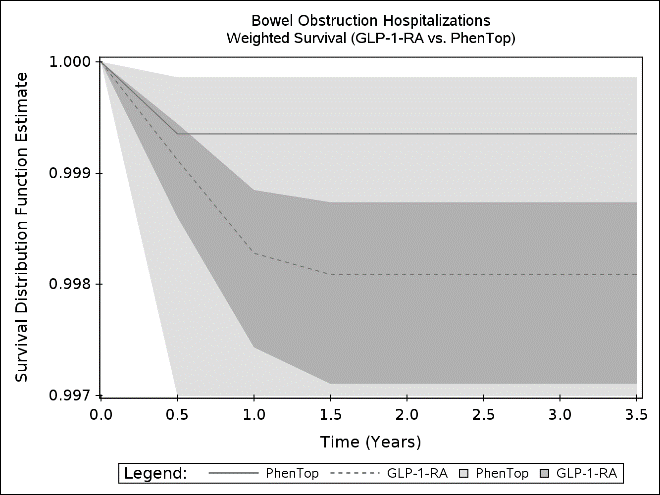

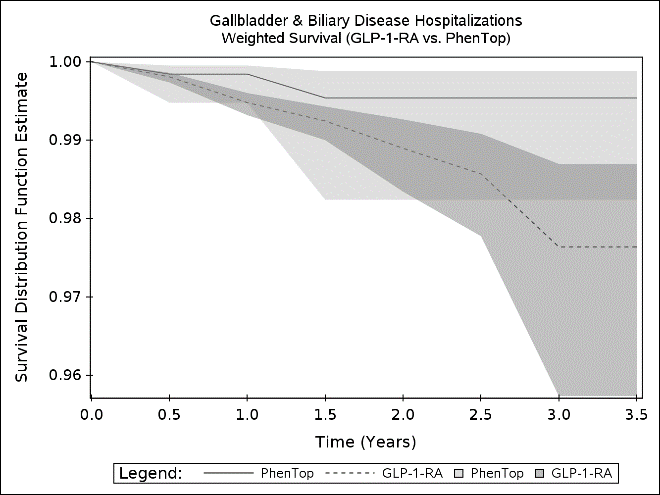

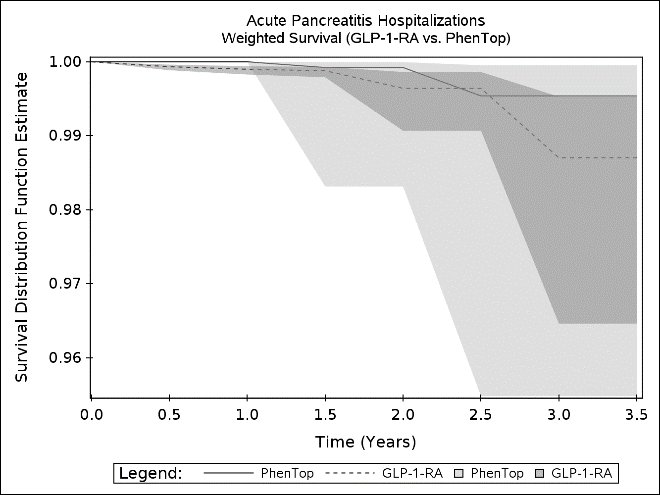

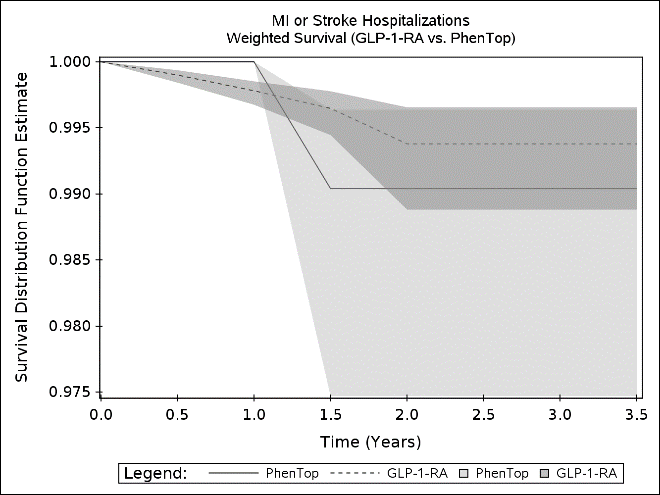

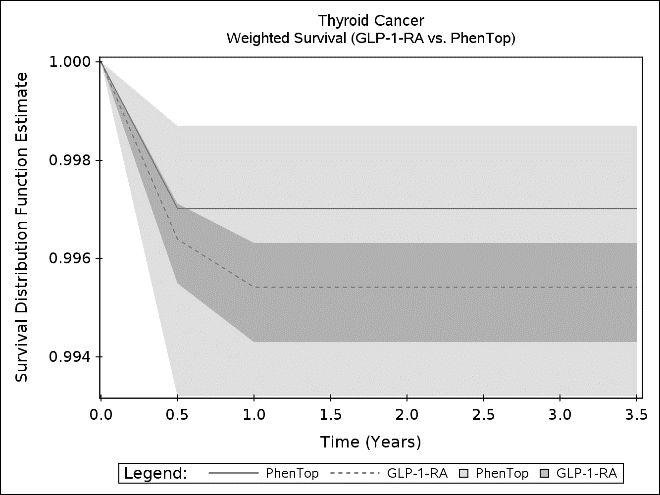

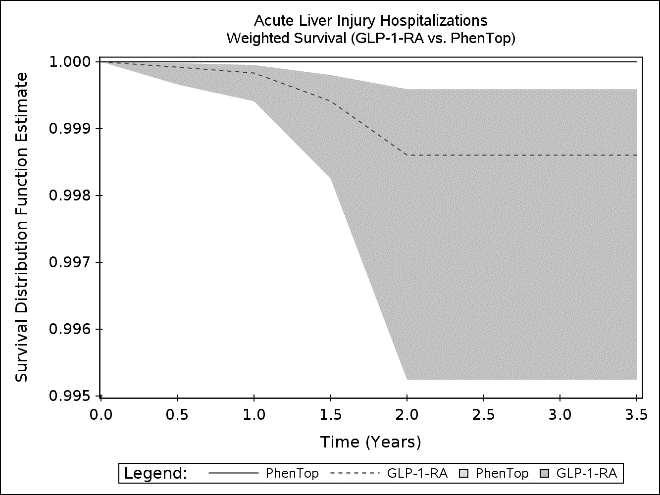

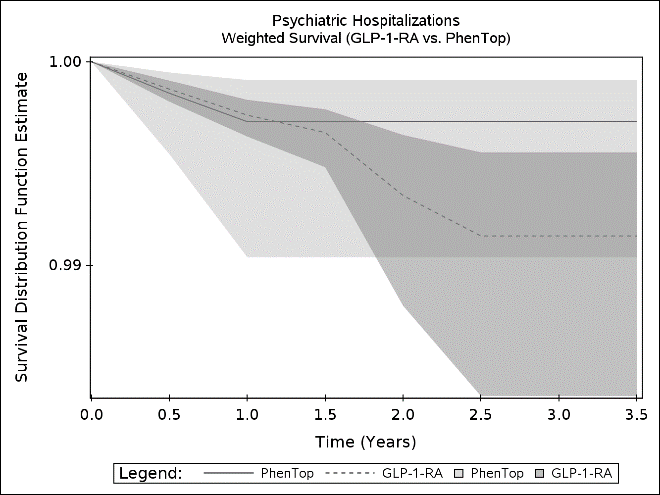

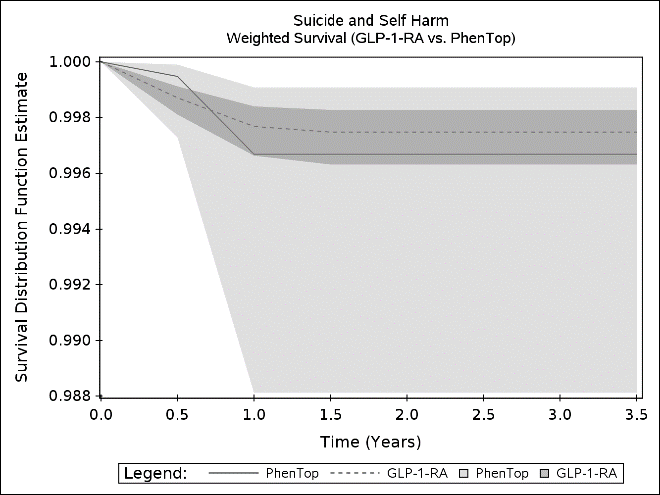

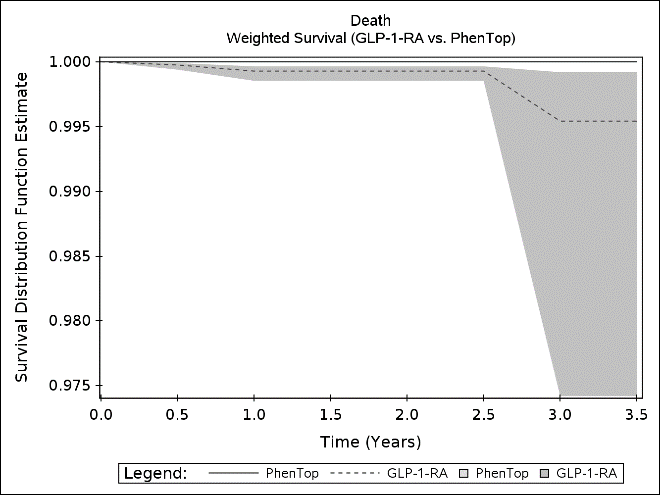

Supplement: Supplementary file 1 — Data S1: Supporting Information. [file PDS-34-e70214-s001.docx]
